# Benchmarking causal reasoning algorithms for gene expression-based compound mechanism of action analysis

**DOI:** 10.1186/s12859-023-05277-1

**Published:** 2023-04-18

**Authors:** Layla Hosseini-Gerami, Ixavier Alonzo Higgins, David A. Collier, Emma Laing, David Evans, Howard Broughton, Andreas Bender

**Affiliations:** 1 Department of Chemistry, Centre for Molecular Informatics, Cambridge, UK; 2 Eli Lilly and Company, Indianapolis, IN USA; 3 Eli Lilly and Company, Bracknell, UK; 4 Centre de Investigación, Eli Lilly and Company, Alcobendas, Spain; 5 Present Address: GSK, Stevenage, UK; 6 Present Address: DeepMind, London, UK; 7 Present Address: Ignota Labs, London, UK; 8 Present Address: Social, Genetic and Developmental Psychiatry Centre, IoPPN, Kings’s College London, London, UK; 9 Genetic and Genomic Consulting Ltd, Farnham, UK

**Keywords:** Transcriptomics, Causal reasoning, Mechanism of action, Network Biology, Benchmarking, L1000

## Abstract

**Background:**

Elucidating compound mechanism of action (MoA) is beneficial to drug discovery, but in practice often represents a significant challenge. Causal Reasoning approaches aim to address this situation by inferring dysregulated signalling proteins using transcriptomics data and biological networks; however, a comprehensive benchmarking of such approaches has not yet been reported. Here we benchmarked four causal reasoning algorithms (SigNet, CausalR, CausalR ScanR and CARNIVAL) with four networks (the smaller Omnipath network vs. 3 larger MetaBase™ networks), using LINCS L1000 and CMap microarray data, and assessed to what extent each factor dictated the successful recovery of direct targets and compound-associated signalling pathways in a benchmark dataset comprising 269 compounds. We additionally examined impact on performance in terms of the functions and roles of protein targets and their connectivity bias in the prior knowledge networks.

**Results:**

According to statistical analysis (negative binomial model), the combination of algorithm and network most significantly dictated the performance of causal reasoning algorithms, with the SigNet recovering the greatest number of *direct targets*. With respect to the recovery of *signalling pathways*, CARNIVAL with the Omnipath network was able to recover the most informative pathways containing compound targets, based on the Reactome pathway hierarchy. Additionally, CARNIVAL, SigNet and CausalR ScanR all outperformed baseline gene expression pathway enrichment results. We found no significant difference in performance between L1000 data or microarray data, even when limited to just 978 ‘landmark’ genes. Notably, all causal reasoning algorithms also outperformed pathway recovery based on input DEGs, despite these often being used for pathway enrichment. Causal reasoning methods performance was somewhat correlated with connectivity and biological role of the targets.

**Conclusions:**

Overall, we conclude that causal reasoning performs well at recovering signalling proteins related to compound MoA upstream from gene expression changes by leveraging prior knowledge networks, and that the choice of network and algorithm has a profound impact on the performance of causal reasoning algorithms. Based on the analyses presented here this is true for both microarray-based gene expression data as well as those based on the L1000 platform.

**Supplementary Information:**

The online version contains supplementary material available at 10.1186/s12859-023-05277-1.

## Introduction

Following their administration in a biological system, many small molecule compounds treat disease by modulating the activity of signalling networks and pathways *via* (direct and indirect) interactions with protein targets [1]. The modulation of targets, signalling proteins and biological pathways describe the mechanism of action (MoA) of such compounds. Previous reviews have highlighted the importance of understanding compound MoA to guide drug discovery [2]—not only to validate observed phenotypic effects, but to understanding side effects [3], identify opportunities for personalised medicine [4], and to repurpose approved drugs for new indications [5]. The complex nature of a compound’s MoA, and the fact that it can be defined by different layers of biology, means that for uncharacterised compounds the elucidation of its MoA is generally a significant bottleneck. To this end, chem- and bioinformatics approaches, based on different types of bioactivity and “-omics” data (such as genomics, proteomics, metabolomics, and—of direct relevance for the current work—transcriptomics), have become popular for generating testable hypotheses by harnessing experimental data with mathematical and statistical analyses and computational algorithms [6].

Recently, large -omics databases have become available in the public domain, such as the LINCS L1000 database [7] (scale-up of the Connectivity Map database [8] with a more high-throughput platform) which catalogues the transcriptional response of a variety of cell lines to treatment with ~ 30,000 different small molecules by measuring a reduced representation of the transcriptome (978 genes) and inferring the expression levels of the remaining genes from this so-called “landmark” set. Although these large transcriptomics data sets provide a rich *starting point* for the understanding of drug mechanisms, a key question is *how* such data should be exploited and interpreted in a meaningful way to shortlist targets or pathways for experimental validation. Differentially expressed genes (DEGs) can be analysed with pathway enrichment methods which calculate the statistical significance of the association of their protein products with annotated biological pathways and processes [9]. Although this approach provides a simple way to reduce large gene sets down to a smaller set of biologically interpretable pathways, it relies on the only partially true association of gene expression with protein activity and abundance [10]. In fact, protein activity is dictated not only by transcription, but translation and post-translational modifications such as phosphorylation—differential gene expression has indeed been found to reflect the activity of upstream transcription factors rather than the activity of a pathway of interest [11].

Methods known collectively as “causal reasoning” have been developed with the aim of identifying causal molecules (of an observed response) by treating differential gene expression as a *consequence* of differential protein activity, rather than equating gene expression with signalling protein activity. Such methods maximise the biological information gained with transcriptomics data by incorporating prior knowledge networks (PKNs) of signed and directed (i.e., *X* inhibit*s Y*) protein–protein interactions (PPIs) to trace upstream of mRNA regulation to the targets and signalling proteins modulated by a compound. Case studies of mechanism of action elucidation using causal reasoning include the elucidation of key processes involved in a DGAT1 inhibitor for obesity [12], and an AKT inhibitor for cancer [13], with the former study remarking that the inferred signalling proteins represented less high-level and more detailed processes in contrast to the findings derived from traditional pathway enrichment methods.

One previous benchmarking study [14] compared several computational network algorithms, finding that the causal reasoning algorithm SigNet [15] (also considered in our study) performed relatively well at recovering target proteins from compound-perturbed gene expression data (from the Connectivity Map dataset [8]), ranking in the top 5 of 17 algorithms in ~ 35% of cases, in terms of fraction of direct targets recovered. The authors did however not consider the impact of prior knowledge network, and neither did they investigate the applicability domain with respect to the nature of protein classes and network connectivity bias and only considered direct targets. Although protein targets are one way to define compound mechanism of action [16], the inference of particular targets from gene expression data is a difficult task due to both conceptual and practical limitations. On the conceptual side, not all target modulations lead to downstream effects in gene expression [17], and gene expression data is downstream of the modulation of often multiple targets [18]. On the practical side, there are high levels of noise in both gene expression data [19] and biological networks [20], and bioactivity datasets are sparse (92% sparsity according to a previous study of PubChem and ChEMBL data [21]). Adding further complexity, compound-protein interactions, protein–protein interactions and pathways are context-specific yet prior knowledge networks are global. In general, there are few benchmarking studies in this field due to this complexity and lack of ground truth data, hence more benchmarking studies are required [22].

## Current study

This study aims to benchmark casual reasoning algorithms for their ability to recover compound mechanism of action (on both the target- *and* pathway-level) from gene expression data, in particular with respect to the following parameters which we anticipated would present the greatest influence on the results, and which had not yet been investigated in previous benchmarking studies (Table 1):

- Algorithm scoring methodology (CausalR (Ranked Table), CausalR (ScanR), SigNet, CARNIVAL).
- Source of input gene expression data (CMap or LINCS, MCF7 or PC3 cell line).
- Input gene subset (landmark genes, landmark and best inferred, or all).
- Prior knowledge network (smaller Omnipath network vs. 3 larger MetaBase™ networks).
- Properties and biological functions of protein nodes (connectivity on network, protein class).

**Table 1:** Parameters considered in this benchmarking study, their specific values under investigation, and the scientific aims of investigating each parameter

Parameter	Aim of investigation
Algorithm	Determine which algorithms perform better, examine how they handle network bias
Network	Examine effects of coverage vs. potential noise
Platform	Comparison between two transcriptomics technologies, evaluation of use of L1000 data with causal reasoning
Gene Set	Evaluation of the ability of reduced representation of transcriptome to gain MoA insights, comparison between inferred vs. measured genes
Cell Line	Evaluate effect of biological context on the ability of the algorithms to recover diverse MoAs

To quantify the performance of the algorithms, we assessed the ability of each algorithm, in combination with different networks and parameter settings, to recover known targets directly, as well as compound-associated pathways (those annotated with the known targets) using a two-step enrichment approached described in the CARNIVAL study [23]. We obtained known compound targets from ChEMBL [24], RepurposingHub [25] and Connectivity Map [8] to allow us to evaluate the output of the algorithms. Drugs also act *via* other mechanisms of action not considered here (e.g., broad DNA damaging agents which don’t bind to a specific protein). Such mechanisms are out of scope of validation in this benchmarking study, which is focused on small molecules with direct protein target(s). It is also important to note that annotated targets may not directly relate to compound efficacy, for example in the case of off-targets, and in vitro activities in particular may not be relevant for in vivo mechanism of action (for example due to PK; and indeed, cancer cell lines may not necessarily reflect in vivo gene expression response), however for the purpose of this study we use target annotations as the best proxy for mechanism of action which is currently available in the public domain for a large-scale benchmark study.

Following the computation of both evaluation metrics, we used a negative-binomial model to understand the contribution of each parameter (input data, network, algorithm) to the recovery of direct targets. Additionally, we investigated the ability of the algorithms to recover informative pathways, and evaluated the applicability domain of the methods by investigating any potential association between the successful recovery of a particular target class and its connectivity in the prior knowledge networks, or its biological role.

Hence, the overall aim of this study was to systematically investigate the ability of causal reasoning algorithms to infer compound mechanism of action on both the target- and pathway-levels, with respect to both the key factors which influence their quantitative performance as well as for which targets/signalling proteins the methods are likely to be more or less successful.

## Materials and methods

### Causal reasoning algorithms

In terms of causal reasoning algorithms, we aimed to see how they handled bias in biological networks, and if this behaviour differed across the different methods. Different causal reasoning algorithms have been implemented in open source and software packages and each requires a specific type of input data (transcriptional response) and PKN (Table 2). Nodes on a prior knowledge network can be prioritised or ranked in a number of ways; for example, by simply counting the number of concordant interactions each node makes with the observed changes in gene expression, and applying a significance calculation to each score (CausalR) [26]. Other methods score network nodes by incorporating gene fold-change statistics (SigNet) [15], or by computing the Kullback–Leibler divergence (relative entropy) of interacting genes in the network based on the differential expression of each measured gene (DeMAND) [27]. Another algorithm additionally uses ODE (ordinary differential equation) kinetic approximations of mRNA regulation to estimate the ability of each node on the network to modulate gene regulatory activity (ProTINA) [28]. As well as ranking network nodes, causal reasoning methods can output subnetworks which capture dysregulated signalling cascades—such subnetworks can be optimised using inferred transcription factor activities and pathway weights, and optionally known bioactivity e.g. protein targets (CARNIVAL) [23], or from connecting nodes of interest (e.g. highly ranked nodes) to input genes via their concordant interactions (CausalR [26]). A comprehensive review of different algorithms can be found in a recent paper by Garrido-Rodriguez et al. [22]. In this benchmarking study, we investigated the CausalR [26] (both node ranking and ScanR subnetwork outputs), CARNIVAL [23] (subnetwork output) and SigNet [15] (node ranking) methodologies. These methodologies were chosen for two reasons, firstly because they require the same input data, so they can be fairly compared. Furthermore, the algorithms are computationally intensive especially over large networks, and we wanted to benchmark other parameters as well. This required us to choose a small enough set of algorithms to be computationally feasible, but still diverse in how they prioritise nodes.

**Table 2:** Summary of commonly used open-source causal reasoning algorithms and whether they were included in this study

Algorithm	Description	Input(s)	Output(s)	Included
CausalR	Nodes scored by counting the number of concordant/discordant interactions Nodes scores are also assessed for statistical significance, returning a p-value	Gene/Protein Z-scores or fold-changes Any signed and directed network	Ranked table of scored nodes with p-values Sub-network of consensus regulators (ScanR) and their concordant interactions	Yes
SigNet	Ensemble of scoring methods which takes into account log fold-changes	Gene Z-scores or fold-changes Any signed and directed network	Ranked table of scored nodes	Yes
CARNIVAL	Integer Linear Programming optimisation of a sub-network capturing signalling changes	Gene/Protein Z-scores or fold-changes Any signed and directed network	Optimised sub-network	Yes
DeMAND	Scores nodes by computing Kullback–Leibler divergence of interacting genes in the network	Treatment and control—level gene expression data Any signed and directed network	Ranked table of scored nodes	No—Different input data required
ProTINA	ODE (ordinary differential equation) kinetic approximations of mRNA regulation to estimate ability of each node on the network to modulate gene regulatory activity	Gene Z-scores or fold-changes Cell-specific network	Ranked table of scored nodes	No—Different input network required

### Gene expression data

The input gene expression data used in this study were from the publicly available Connectivity Map (CMap [8]) and LINCS L1000 [7] gene expression databases, which are often used in bioinformatics approaches for MoA elucidation [29]. CMap contains microarray data for ~ 1300 compounds, and is no longer updated. The LINCS L1000 database is a scale-up of the CMap database, and instead of microarrays the high-throughput L1000 platform uses a Luminex bead-based system to measure the expression levels of 978 “landmark genes” chosen to be a reduced representation of the transcriptome. The database also contains predicted expression values for around 12,000 other genes, of which a subset is denoted as “best inferred genes”, i.e., they were found to be predicted correctly most often. Compounds under investigation under this study were selected based on their presence in both databases, measured in the MCF7 and PC3 cell lines. For the purpose of benchmarking CMap versus LINCS specifically, a large overlap of compounds in both data sets was required, and hence we could only use data from MCF7 and PC3. This is because these two cell lines are the only that have a substantial amount of data measured in CMap [8] and also are the 2nd and 3rd most data-rich cell lines in LINCS [30]. Additionally, PC3 and MC7 are frequently chosen as a subset of cell lines in previous investigations of the LINCS data due to the large amount of compounds screened [31, 32].

By comparing L1000 with its predecessor, CMap, we aimed to disentangle any effects arising from the *quantity* of input genes (i.e., using 978 instead of 10,000+) from the effects arising from the *quality* of poorly-predicted non-landmark genes, as well as any differences in technologies between the two platforms. Hence, for each compound measured under particular conditions (cell line, time point, dose), we obtained three signatures forming three different input gene sets—consisting of landmark genes only (n = 987), “best-inferred” genes (n = 10,174), and all genes (n = 12,329). Landmark genes were selected in a data-driven way rather than for biological discovery[7], hence the ability of these genes to recover MoAs from causal reasoning is of interest. Although compound targets will not all be present in the list of L1000 genes, their downstream genes (directly or indirectly modulated by the target) may be. It is hence plausible that causal reasoning algorithms are able to use interaction networks to infer upstream targets (if present in the network), despite not being present in the landmark gene list. Though the MoAs present in the dataset were diverse (Additional file 1: Fig. 1), and not necessarily within the biological context of PC3 and MC7 cells (e.g., neuronal pathways), we wanted to explore whether or not this presented a limitation in practice.

### Prior knowledge annotations

We also evaluated whether different algorithms handled increasing network size and density in a different manner, owing to the differences in how they prioritise causal proteins, and to understand whether performance scales with network size; or rather if added noise is detrimental. There are many sources of publicly available protein–protein interaction data all containing different information of varying levels of completeness and confidence [29], so in this study we chose to use the Omnipath database which contains a curated combination of 44 different sources of protein–protein interaction information [33]. As a comparator we also used the MetaBase™ commercially available networks which are much larger (more nodes) and denser (more connections) than publicly available databases (Additional file 1: Table 1), and annotate each interaction with a confidence score assigned by a curator. We derived three different networks from MetaBase; denoted herein as high, medium and low confidence networks. The high confidence network contained only interactions annotated as high confidence, medium contained high and medium confidence interactions, and low contained all interactions including low confidence interactions.

For pathway annotations (used to assess the output of causal reasoning algorithms), we used the Reactome knowledgebase because it is generally considered to be high-quality (due to manual curation efforts [34]), covers a wide range of biological processes (2,601 human pathways covering 11,097 proteins under 20 core processes such as Cell Cycle, Metabolism, etc. as of December 2022 [35]). In particular is laid out in a hierarchical structure (where child pathways are increasingly more granular) which made it possible to quantify how specific or general the recovered pathways were. There are many other sources of pathway annotations, e.g., KEGG [36], WikiPathways [37], which contain fewer unique pathways, and it has been found that the choice of pathway knowledgebase influences the results of statistical enrichment analysis [38]. It is recommended, for a real-life use-case, that researchers explore the available databases [29, 39] and their coverage with respect to e.g., species, biological processes, or for greater coverage use a combination of different databases to perform pathway enrichment [38, 40]. For example, if the study is focused on the liver, to choose a database which contains enough liver-specific processes to adequately represent the biology being modelled (e.g., bile salt synthesis, xenobiotic metabolism), or if the biological mechanism is unknown to combine databases for more coverage of potential pathways. All networks and pathways used in this study are global and are hence not cell- or context-specific.

### Benchmarking set up

**Fig. 1:**
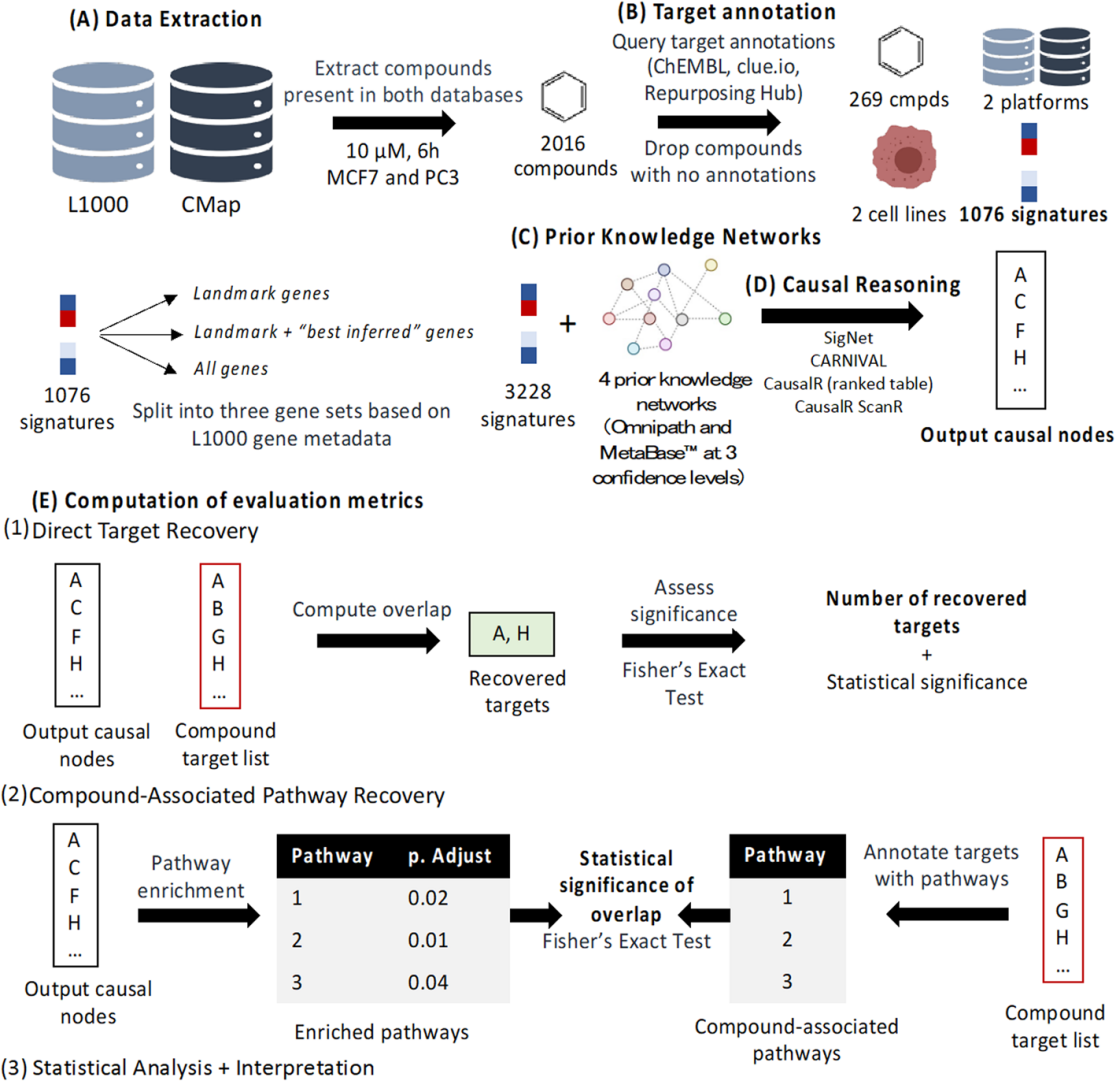
Workflow for the current study, involving extraction of data from L1000 and CMap measured at 10µM and 6 h, in the MCF7 and PC3 cell lines. Compounds were annotated with targets and signatures split into three gene sets based on their identity in the L1000 metadata. Causal reasoning was performed and evaluation metrics computed, on the target- and pathway-levels. Finally, statistical analysis and interpretation of the results were performed

The overall workflow for the benchmarking study can be found in Fig. 1. The workflow involves the extraction and cleaning of gene expression data, querying for target annotations, processing signatures into gene sets, extracting and cleaning prior knowledge networks, application of the causal reasoning algorithms, and finally computation of evaluation metrics on the target- and pathway-levels.

#### Data extraction

All compound data in CMap and LINCS derived at a time point of 6 h after compound treatment and using a compound concentration of 10µM were extracted for this study, measured in the two cell lines common to both datasets (MCF7 and PC3), see Additional file 2. While this (relatively high) concentration is unlikely to represent physiological compound concentrations after drug administration, here only a link on the in vitro level between gene expression signal and compound mode of action was aimed to be established, plus the majority of data was only available at this concentration from the practical angle. Furthermore, early (hit discovery) screening assays are typically run at 10 µM concentrations [8, 41]. The short time-point of 6 h post-perturbation was selected because we assume that at a shorter time-point the direct transcriptional changes induced by the compound are captured, rather than second or tertiary effects that occur over a longer period of time. On that basis, the authors of CMap suggest 6 h to observe direct mechanism of action [8]. This produced a subset of 2,016 unique compounds meeting the specified conditions.

#### Target annotation

Each of the 2016 compounds were queried in the ChEMBL [24], and clue.io [42] RepurposingHub [25] databases to retrieve their known (measured) bioactivities. The bioactivity data were used to annotate each compound with a list of known targets, either labelled in RepurposingHub or with a measured activity of 10 µM or better in ChEMBL (Additional file 2). Compounds with no bioactivity data in either database were dropped, leaving a total of 269 compounds with “ground truth” measurements (Additional file 3). To facilitate the interpretation of the results, each annotated target was labelled with its protein classification in ChEMBL (Additional file 2).

#### Prior knowledge networks

For this study, we benchmarked signed and directed networks from Omnipath and MetaBase™ (Fig. 1C). The Omnipath protein–protein interaction network (9306 interactions) was extracted directly from the Additional file 1 in the CARNIVAL publication (Additional file 2). The MetaBase networks were extracted at three thresholds of confidence; low (all interactions, 87,556), medium (57,758 interactions) and high (51,730 interactions), see Additional file 2. For the SigNet algorithm, additional transcriptional regulatory interactions were obtained from Omnipath and MetaBase (Additional file 2).

#### Causal reasoning pipeline

To convert the data from gene- to protein-level to be used with protein–protein interaction networks (CausalR and CARNIVAL), we first used the DoRothEA regulon with the VIPER statistical method to infer upstream TFs from the expression levels of their gene targets (Additional file 2). This is not a trivial task in itself; however, this method was chosen firstly due to it showing good performance (AUPRC 0.5–0.8 with DoRothEA[43], AUC 0.62 [44]), and because it has a demonstrated use in a variety of applications [45–47] including with the LINCS L1000 data as input [48]. SigNet on the other hand uses transcriptional regulatory interactions to infer TFs from genes as a first step, so the gene-level data was used directly with this algorithm. Furthermore, we used PROGENy to derive pathway activity scores from gene-level data for use with the CARNIVAL algorithm (Additional file 2), as recommended by the authors to aid the network optimisation [23].

Each algorithm (SigNet, CausalR Ranked Table, CausalR ScanR and CARNIVAL) was then applied to the processed data (including a modified ScanR code, all details given in Additional file 2). These algorithms when implemented produced node lists as output, either in the form of subnetworks (CARNIVAL, CausalR ScanR) or ranked tables of nodes ordered by score (CausalR Ranked Table, SigNet). The output nodes were hence used to compute evaluation metrics.

#### Computation of evaluation metrics

The algorithms produced two types of output, subnetworks and ranked lists, as summarized in Table 3. For network outputs (CARNIVAL, ScanR) the full network was assessed, while for CausalR Ranked Table, all nodes with a p-value of < = 0.05 were assessed. As SigNet does not compute significance values for ranked nodes, we computed the mean number of output nodes for the other algorithms; this was found to be 198, hence we used this as the cut-off for the top ranked nodes taken from the SigNet output.

**Table 3:** Algorithm outputs and the evaluation metrics applied in every case

Algorithm	Output	Nodes Assessed
CARNIVAL	Default output subnetwork	Full network
CausalR (ScanR)	ScanR subnetwork	Full network
CausalR (Ranked Table)	RankTheHypotheses table	All nodes with p < = 0.05
SigNet	Default output table (overall score)	Top 198 scored nodes

1. Direct Target Recovery

To evaluate if direct targets were recovered, output from the algorithms was intersected with the known targets of each compound extracted from ChEMBL and RepurposingHub (Fig. 1E). To compute the significance of the number of targets recovered based on the number of compound targets, the number of potential nodes recoverable from the prior knowledge network, and the cardinality of the output, the R function fishers.test(alternative=”greater”) was run. The number of recovered targets per compound were subsequently modelled using statistical analysis. If the target was not present in the prior knowledge network, it was discounted from the significance calculation. Compound outputs where no targets could be recovered (i.e., no targets were present in the network) were discounted from the subsequent statistical analysis. Additional file 1: Table 1 summarises target coverage for each network.

2. Pathway Enrichment.

To quantify recovery of ‘relevant pathways’, we used the same principles as in Liu et al.’s CARNIVAL evaluation metric which examines whether pathways which contain the target of interest are recovered [23], but instead combining *all* target-associated pathways into one set of compound-attributed pathways to take into account polypharmacology. For each compound, each target was annotated with its participating pathways using the ReactomePA [49] R package. The set of unique target-annotated pathways was denoted as the compound-associated pathway set. Secondly, enrichment of output nodes in the Reactome pathway set was performed using the ReactomePA package [49] with all prior knowledge network nodes as the background/universe, and enrichment p-values adjusted with the Benjamini-Hochberg procedure [50]. This led to two pathway lists: Firstly, compound-associated pathways obtained from target annotations (ground truth), and, secondly, significantly enriched (adjusted p-value < = 0.05) pathways obtained from causal reasoning output nodes. To compute the over-representation of enriched pathways in the set of ground truth pathways, we computed a second enrichment p-value using fisher.test(alternative=“greater”) with all Reactome pathways set as the background/universe. We hence interpret the *second* enrichment p-value as the extent to which target-associated pathways were captured in the causal reasoning output.

Furthermore, as Reactome pathways are laid out hierarchically under high-level categories such as “Cell Cycle”, “Metabolism”, “Neuronal System”, we used the position of recovered pathways in the hierarchy, as well as the number of protein annotations, as a proxy for pathway specificity, where higher-level and larger pathway sets were assumed to be less specific and hence less informative for understanding compound mechanism of action. To this end, we downloaded the entire Reactome knowledgebase as a Neo4J object [51]. Following the initialisation of a Neo4J Reactome database, we retrieved the number of superpathways for each Reactome pathway (pathways above the pathway in question in the hierarchy) using the neo4r R package [52], with the command ‘MATCH (p:Pathway{stId:[PATHWAY-ID]})<-[:hasEvent*]-(sp:Pathway) RETURN p.stId AS Pathway, sp.stId AS SuperPathway, sp.displayName as DisplayName’.

We repeated the two-step enrichment analysis with pathways enriched from CMap genes |Log2FC| >= 1.5 and LINCs genes |ZScore| >= 2 using the same methodology. If no pathways were significantly enriched, or if there were no genes with which to perform the enrichment, the second enrichment p-value was set to 1 (no enrichment).

### Statistical analysis

To understand which factors were most influential in recovering compound targets, the number of targets recovered for each compound for every combination of factor levels (following a Poisson distribution, Additional file 1: Fig. 2) were modelled using a Type II negative binomial distribution model (Hardin & Hilbe 2007) and restricted maximum likelihood (REML) estimation using the Generalized Linear Mixed Models using Template Model Builder (glmmTMB) R package. First, a model was built considering all single and two-way interactions using the following function:

glmmTMB(Overlap ~ (Network + Algorithm + Platform + Gene_Set + Cell_Line)^2

+ (1 | Compound), REML = T, family="nbinom2”)

where the (1|Compound) term accounts for repeated observations (as the same compounds were tested under all sets of conditions). A final model was built which included the single effect terms and only the significant (p < = 0.05) interaction effect terms from the first model:

glmmTMB(Overlap ~ Network + Algorithm + Platform + Gene_Set + Cell_Line +

Network*Algorithm + Platform*Gene_Set + (1 | Compound), REML = T, family="nbinom2”)

The final model had a dispersion ratio of 1.010 (p = 0.055) indicating no overdispersion. Post-hoc comparisons were carried out using estimated marginal means implemented in the emmeans R package using the following function:

emmeans(model, ~term, mode = “df.error”, type=”response”)

where “term” is either a significant single effect not appearing in any interaction effects (e.g., Cell Line) or a significant interaction effect (Network + Algorithm). If factors interact in a statistical model, it is generally considered to be not useful to perform post-hoc analysis on the individual factors themselves. This is because in the presence of a significant interaction, any effort to interpret the main effects of the factors involved will be based on the false premise that differences on one factor exist across all levels of the other factor(s) [53]. The estimated marginal means were compared pairwise to find significant differences between them (at a significance level of 0.05) and reported as compact letter displays (CLD) using the sidak method for confidence level and p-value adjustment. This was achieved using the following function from the multcomp R package:

cld(emmeans,alpha = 0.05,adjust=“sidak”)

## Results and discussion

### Target recovery depends on the network and algorithm interaction effect

To understand which factors are most important when employing causal reasoning algorithms to recover compound targets, we modelled the target overlap evaluation metric (which follows a Poisson distribution of count data, Additional file 1: Fig. 2) as a dependent variable and the parameters as predictors (network, algorithm, platform, gene set, and cell line) in a Type II negative binomial model. The full model parameters are given in Additional file 1: Tables 2 and a truncated version of the results with each term’s p-value is given in Table 4. The most significant (p < 0.001) terms were the Network:Algorithm interaction effect and the Platform: Gene Set interaction effect (specifically the LINCS platform and landmark genes, p = 0.0003). PC3 cell line was also a significant term (p = 0.007) not appearing in any interaction effects. Hence, to understand the practical consequences of these findings when using causal reasoning algorithms to recover mechanistic targets, the Network:Algorithm and Platform:Gene Set interaction terms and the Cell Line single term were investigated further with post-hoc pairwise comparisons of least-square means.

**Table 4:** Terms and probabilities (indicating the significance of the effect) for the negative binomial model of number of targets recovered by causal reasoning

Term	p-value
NetworkMetabase_High	1.87e-11
NetworkMetabase_Med	2.27e-10
NetworkOmnipath	< 2e-16
AlgorithmCausalR_Network	< 2e-16
AlgorithmCausalR_RT	< 2e-16
AlgorithmSigNet	< 2e-16
PlatformLINCS	0.248235
Gene_SetBING	0.295338
Gene_SetLM	3.06e-06
Cell_LinePC3	0.007366
NetworkMetabase_High:AlgorithmCausalR_Network	4.38e-13
NetworkMetabase_Med:AlgorithmCausalR_Network	1.85e-12
NetworkOmnipath:AlgorithmCausalR_Network	< 2e-16
NetworkMetabase_High:AlgorithmCausalR_RT	7.15e-13
NetworkMetabase_Med:AlgorithmCausalR_RT	1.46e-08
NetworkOmnipath:AlgorithmCausalR_RT	< 2e-16
NetworkMetabase_High:AlgorithmSigNet	3.77e-08
NetworkMetabase_Med:AlgorithmSigNet	1.32e-07
NetworkOmnipath:AlgorithmSigNet	< 2e-16
PlatformLINCS: Gene_SetBING	0.321868
PlatformLINCS: Gene_SetLM	0.000346

**Fig. 2:**
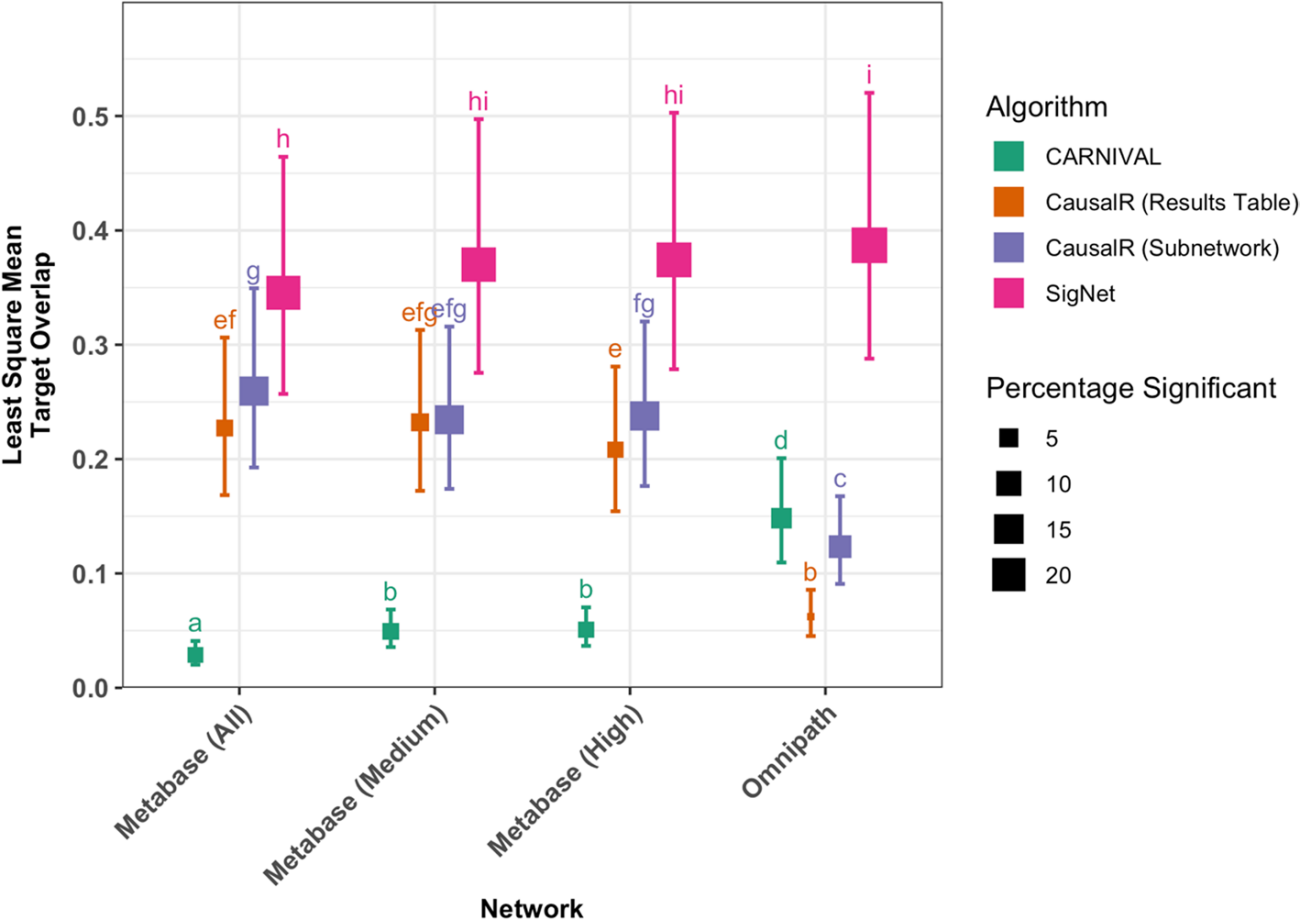
Interaction plot showing post-hoc least square means across all other factors for the Network:Algorithm interaction effect from the negative binomial model of the target recovery evaluation metric. Means sharing a letter are not significantly different according to pairwise comparisons of least square means, with Sidak adjusted p-values for multiple comparisons. Error bars indicate the least square means Sidak 95% confidence interval. The percentage of cases where the target overlap was found to be significant (p < = 0.05, Fisher’s Exact Test) is encoded in point size

After performing post-hoc analysis it was found that the Platform:Gene set and Cell Line effect terms, though statistically significant in the model, did not lead to practically relevant changes in terms of least-square mean response (Additional file 1: Figs. 3 and 4) and hence these were not investigated further. The Network:Algorithm interaction effect however showed that different algorithms behaved markedly differently with each network. From the post-hoc interaction plot in Fig. 2 (also tabulated in Additional file 1: Table 3) it can be seen that the use of the smaller, less dense Omnipath network led to a higher mean target recovery for both the CARNIVAL (0.15 targets per compound, 6.14% significant) and SigNet (0.39 targets per compound, 23.36% significant) algorithms compared to using the larger MetaBase™ networks (for each of “High”, “Medium” and “All” confidence levels), in terms of both the overlap and the percentage of cases where this overlap was significant (p < = 0.05, Fisher’s Exact Test, when considering the number of targets, size of network and number of nodes recovered). While SigNet retained the highest percentage of significant (> 20% for all networks), the CARNIVAL-recovered direct targets were more statistically significant with Omnipath compared to the MetaBase™ networks (6.14% vs. 3.21 and 3.4%). Conversely, the two CausalR algorithms recovered more compound targets directly with the larger, denser MetaBase™ networks, achieving a mean target overlap of just 0.062 (CausalR Results Table) and 0.12 (CausalR Subnetwork) targets per compound with Omnipath, with the percentage significant cases also decreasing.

The improved performance of CARNIVAL with Omnipath compared to the larger MetaBase™ networks for recovering direct targets is likely related to CARNIVAL being optimised using the same Omnipath network used in this study [23]. The results also indicate that SigNet tends to highly rank true positives using the smaller Omnipath network, whereas CausalR works more effectively when there are more interactions to “reason” over. This is in contrast to previous literature which suggests that the potential negative effects of noise are outweighed by a comprehensive inclusion of interactions when using biological networks as prior knowledge for node prioritisation algorithms [54, 55]. In fact, we can conclude from our analysis that—when using causal reasoning algorithms to prioritise compound targets—this behaviour is wholly dependent on the algorithm being used; the CausalR algorithms do benefit from a large prior knowledge network, whereas SigNet and CARNIVAL recover compound targets more effectively with the smaller Omnipath network which contains less potential noise or false positive interactions. Overall, the number of targets recovered was relatively low, which indicates that causal reasoning algorithms are not often able to recover mechanistic targets. This is also supported by the distribution of counts in Additional file 1: Fig. 2 which shows that most of the time, no direct targets were recovered.

### Pathway enrichment using causal reasoning-derived nodes improves on using differentially expressed genes

**Fig. 3:**
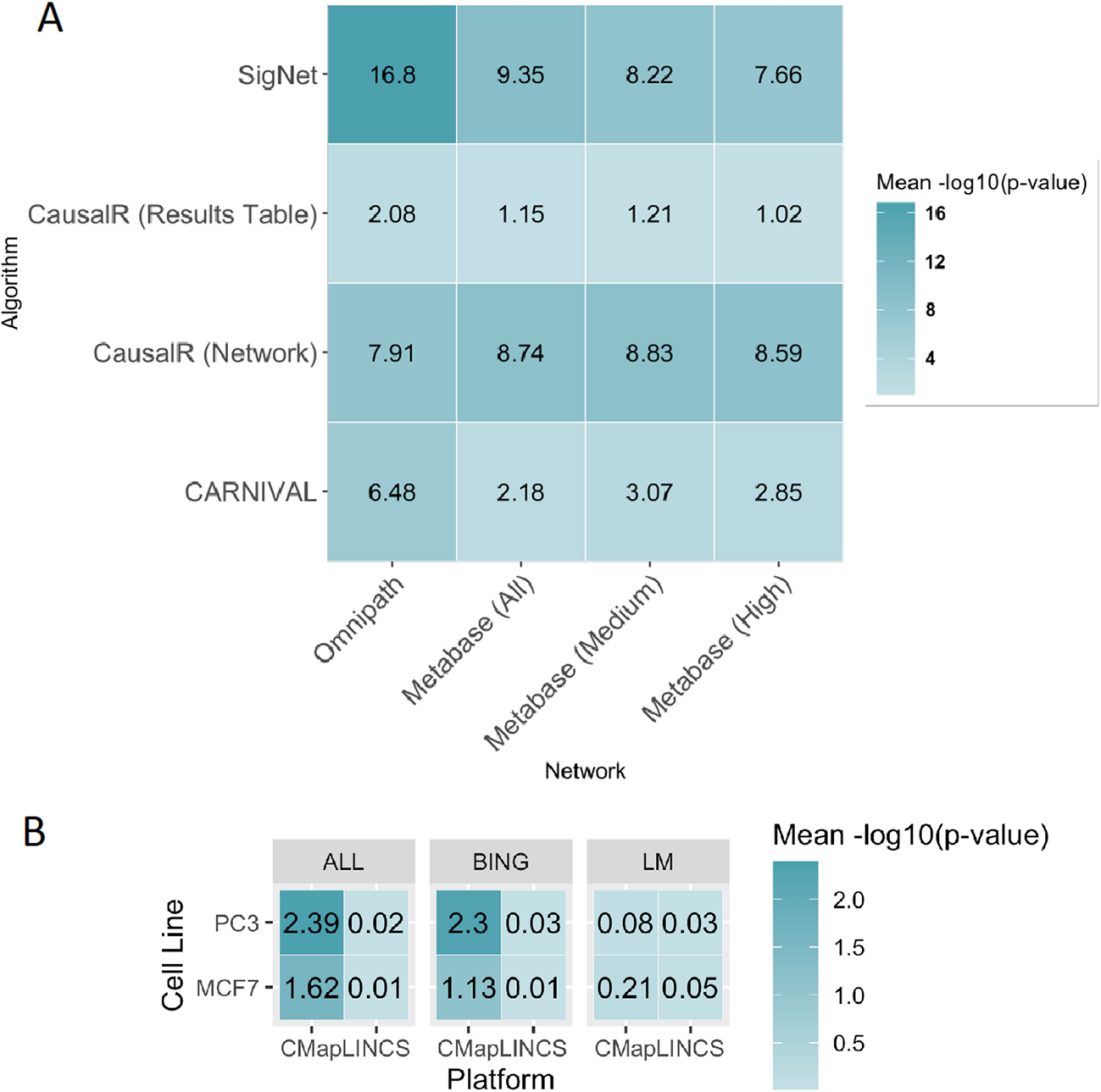
Mean − log10(p-value) of the enrichment in compound target-associated pathways for enriched pathways derived from **A** causal reasoning nodes, for combinations of networks and algorithms and **B** differentially expressed genes, for combinations of cell lines, gene sets and platforms. A higher value indicates a greater significance of the overrepresentation of compound target-associated pathways

Next, we wanted to understand how networks and algorithms impacted the performance of causal reasoning when recovering signalling proteins in pathways known to contain compound targets. The evaluation metric under investigation was the two-step enrichment results where enriched pathways from causal reasoning output were compared for their over-representation in pathways annotated to compound targets in terms of the Fisher’s Exact Test p-value. This metric hence represents the ability of the algorithms to recover signalling proteins involved in mechanistic pathways containing the true compound target(s). To aid in the comparisons, p-values were transformed to their − log10(p-value) and the mean found for each combination of network and algorithm. The results are plotted as a heatmap in Fig. 3A. Because pathway enrichment is typically performed with gene expression data, we also performed the same analysis instead comparing pathways enriched from differentially expressed genes with compound target-associated pathways. We again plotted the mean − log10(p-value), this time for each combination of gene platform, cell line and gene set. This provided baseline results to compare the causal reasoning results with (Fig. 3B).

As can be seen from Fig. 3A, pathways derived from causal nodes from SigNet with the Ominpath network had the best mean enrichment (− log10(p-value)) of compound-associated pathways, with an enrichment value of 16.8, with the worst overall performance seen for the CausalR results table (and enrichment values between 1.02 and 2.08). The CausalR network output achieved a better performance over the results table (enrichment values between 7.91 and 8.83). Like SigNet, CARNIVAL showed a significantly higher performance (enrichment value of 6.48) with the Omnipath network compared to the MetaBase™ networks (enrichment values of 2.18, 2.85 and 3.07 for All, High, and Medium, respectively).

The best enrichment results using differentially expressed genes had a mean of 2.39 (for CMap, PC3 cell line, all genes, Fig. 3B), which was improved on by CausalR Network, SigNet and CARNIVAL (except for when the full MetaBase™ network was used). The CausalR results table results are roughly similar to the CMap gene expression results, which produced mean enrichment values ranging from 1.13 to 2.39. Pathway enrichment using the L1000 data and only considering landmark genes had the worst overall results, which ranged on average from 0.01 to 0.21, below the typical significance cut-off of p = 0.05 (which corresponds to a − log10(p-value) of around 1.3). These results indicate that the causal reasoning algorithms were as good as (CausalR results table) or better (SigNet, CARNIVAL, CausalR network) able to recover relevant signalling proteins compared to using differentially expressed genes alone as a proxy for modulated signalling mediators. This in agreement with Liu et al.’s findings that CARNIVAL outperformed DEG enrichment for recovering relevant pathways [23], and suggests that to understand which pathways a compound is acting on, causal reasoning should be performed instead of traditional pathway enrichment using differentially expressed genes as input.

### Pathways derived from causal reasoning are informative and specific

As well as considering the enrichment p-value as a performance measure, we next aimed to understand how informative, and hence practically useful for understanding compound mechanism, the recovered pathways were. Reactome pathways are laid out in several hierarchies, consisting of the high-level pathway term (e.g., *Cell Cycle*) which contains several sub-pathways which are more specific processes (e.g., *Cell Cycle, Mitotic)*, which themselves have sub-pathways (e.g., *S Phase*), and so on. To quantify how informative recovered pathways were, we assumed that pathways which are lower down in the Reactome pathway hierarchy, and that contain fewer genes/proteins, are more specific and informative for MoA understanding than higher-level, larger, general pathways.

**Fig. 4:**
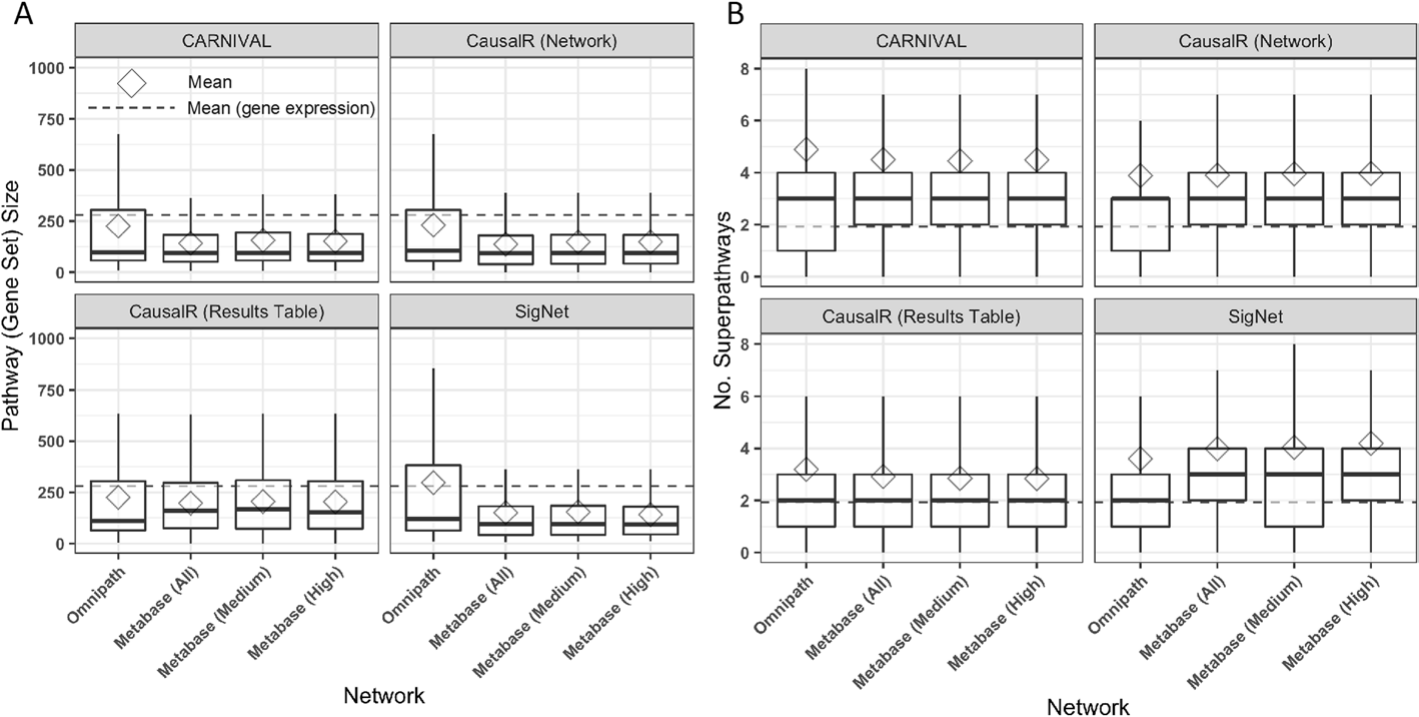
**A** Boxplot of Reactome pathway (gene set) sizes (number of attributed genes/proteins) for the correctly recovered pathways with baseline gene expression results indicated with a dashed red line—smaller is better. **B** Boxplot of number of superpathways within the Reactome hierarchy for the correctly recovered pathways with baseline gene expression results indicated with a dashed red line—larger is better

Boxplots for each combination of network and pathway for recovered pathway size (number of attributed proteins/genes), and hierarchy (number of superpathways), can be seen in Fig. 4A and B, respectively. It was found that, despite the good performance in terms of statistical significance, SigNet with Omnipath pathways were both larger (mean = 300 genes) and higher up in the Reactome hierarchy (mean = 3.6 superpathways) compared to the MetaBase™ networks (mean gene set size below 160, number of superpathways greater than 4). CARNIVAL also recovered pathways that were larger (mean = 225 genes), but lower down in the Reactome hierarchy (mean = 4.9 superpathways, the highest mean value overall), with Omnipath compared to the MetaBase™ networks (mean gene set size below 160, number of superpathways ~ 4.5). Pathways recovered by the CausalR results table were the least informative, with the lowest mean number of superpathways, while the CausalR subnetwork recovered pathways had better results in comparison (smaller mean gene set sizes and greater mean number of superpathways), showing that the ScanR subnetwork methodology is superior to the results table when using CausalR to retrieve compound-associated pathways.

Other than with respect to average pathway size for SigNet with Omnipath, all combinations of network and algorithm recovered more informative pathways compared to average baseline gene expression results (mean gene set size = 280, number of superpathways = 1.9), indicating that pathways recovered from DEGs generally capture higher-level processes compared to causally inferred proteins. This was also found in an application of causal reasoning to understand the mechanisms of a DGAT1 inhibitor, where the authors found that enrichment with gene expression data generally pointed to higher-level processes compared to the knowledge captured by the causally inferred proteins [12]. We hypothesis that this is due to transcriptional changes capturing the *effect* of protein signalling, while causal nodes represent the signalling proteins themselves.

Overall, these results show that performing pathway enrichment with causal reasoning-derived proteins captures more pathways which contain the compound’s direct target, as well as being more informative (describing more specific rather than general biological processes), compared to using differentially expressed genes.

### Causal reasoning is influenced by network bias and biological function

We next investigated performance of the algorithms as a function of the connectivity of a protein target in the network, due to the known connectivity bias present in biological networks [56], as well as its biological function.

**Fig. 5:**
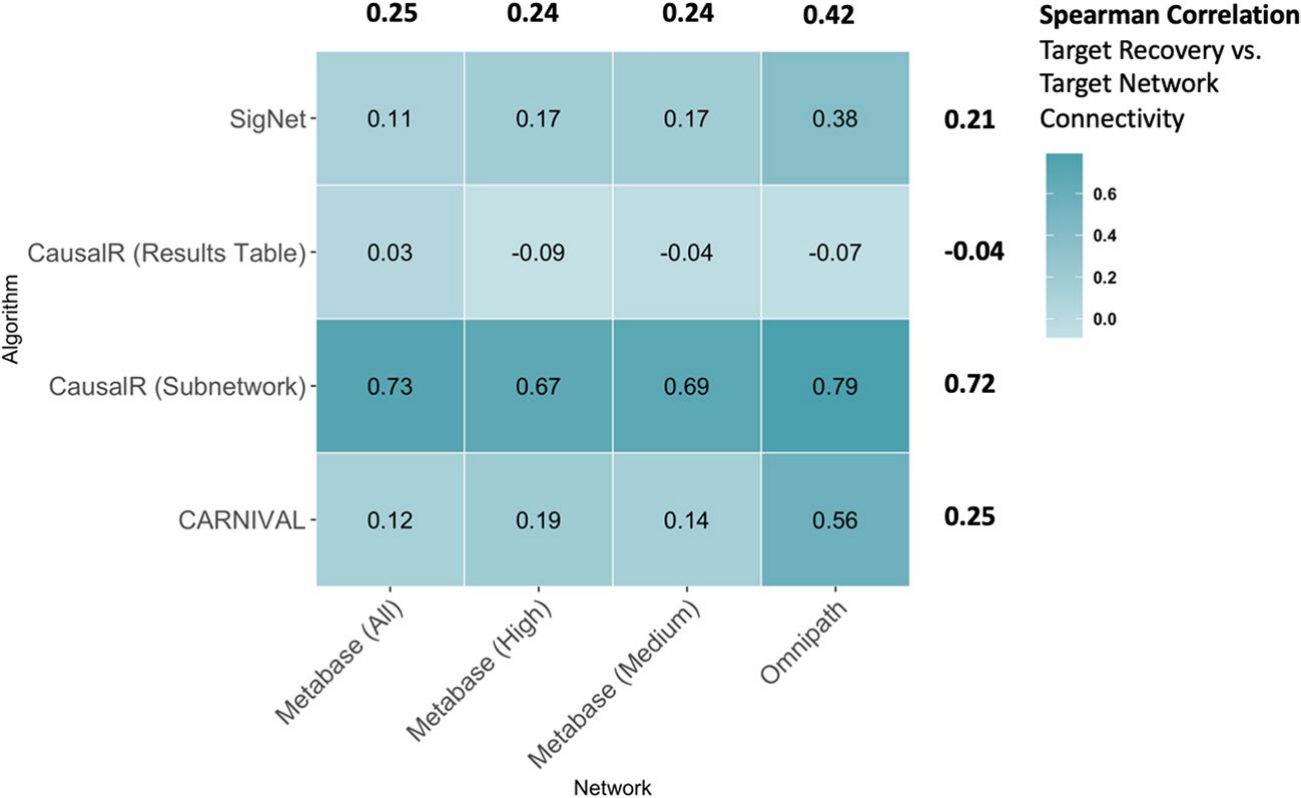
Heatmap showing the Spearman rank correlation of target recovery versus target connectivity on the prior knowledge network, for each combination of network and algorithm. Mean correlation values for each network (top) and algorithm (right) are also shown

For each combination of network and algorithm we calculated how many times each target was recovered. We then normalised this value to account for annotation prevalence by dividing it by the number of times the target was annotated in the compound set. Finally, we calculated the Spearman rank correlation of the normalised target recovery with their degree in the corresponding PPI network (Fig. 5). The lowest correlation can be seen for the CausalR results table (mean of − 0.04) which is likely due to the fact that each protein is given a significance value to explicitly correct for the known connectivity bias, and we take as output only the nodes with p < = 0.05. The correlation between target recovery and network connectivity was highest using the CausalR subnetworks (mean of 0.72)—an explanation for this is that they connect key drivers to input TFs through correctly explained interactions, and will therefore go through “hub” nodes more often. Despite this large difference in correlation, the two CausalR outputs performed roughly similarly in terms of direct target recovery (Fig. 2), which indicates that the CausalR ranked table is better able to prioritise less-connected (and hence less-studied) targets compared to the subnetwork output. SigNet (mean of 0.21) and CARNIVAL (mean of 0.23) showed roughly similar correlation patterns, with the correlation between target recovery and network connectivity increasing (to 0.38 and 0.56, respectively) with use of the Omnipath network, corresponding to their increased performance with this network (Fig. 2). Additionally, we found that targets recovered with the Omnipath network showed a higher correlation with network connectivity (mean of 0.42) compared to the MetaBase™ networks (means of 0.25, 0.24, 0.24), this is potentially due to the small size of the network making hub effects more prominent. Overall, target recovery performance with causal reasoning is generally associated with network connectivity—excluding the targets recovered with the CausalR results table. We further examined the degree distributions of targets vs. non-targets on each network, finding that drug targets were more often found to have a higher connectivity on each network compared to non-targets (Additional file 1: Fig. 6, and generally seen in previous studies [57]). Therefore, a high correlation between target recovery and network connectivity is not necessarily detrimental—however, this bias would affect the recovery of less-studied proteins which must be kept in mind depending on the disease area being studied.

**Fig. 6:**
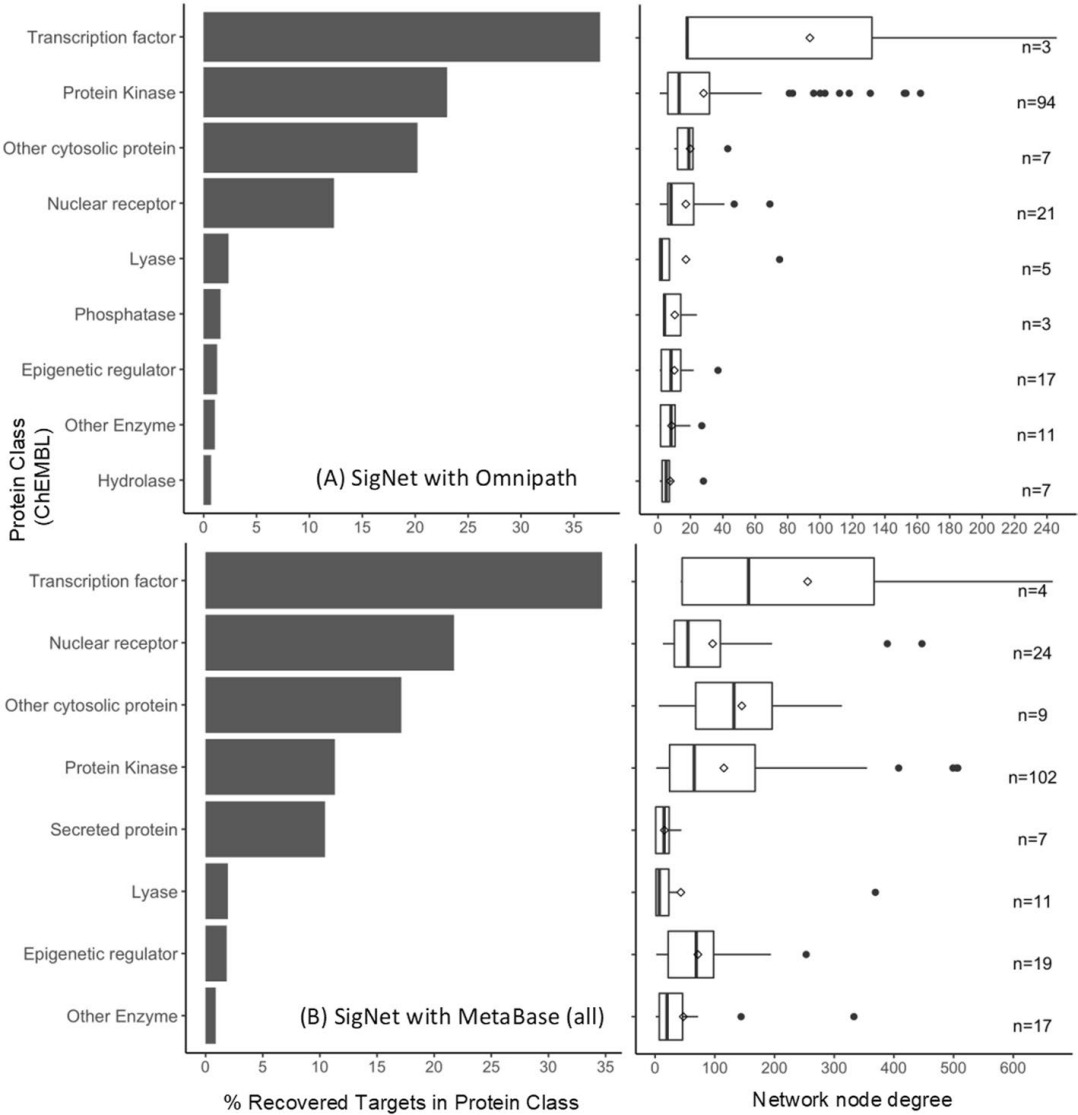
Protein class recovery (% of directly recovered targets in protein class) and network node connectivity for **A** SigNet with Omnipath and **B** SigNet with the full MetaBase™ network. Mean node degrees are represented by a diamond shape in the box plots, while the number of observations is labelled on the right-hand side of the plot

We next analysed the best-performing algorithm (namely, SigNet with the Omnipath network) in more detail with respect to its ability to recover targets across different protein classes (annotations retrieved from ChEMBL, see Additional file 2), comparing the findings with SigNet with the full MetaBase™ network, the results of which are shown in Fig. 6. We computed protein class recovery in the same way as target recovery, calculating how many times a target in protein class was recovered and normalising this value by the annotation prevalence of the protein class in the compound set, converting this value to an overall percentage. Protein classes which had a higher connectivity in the Omnipath network such as transcription factors and protein kinases were recovered more often with SigNet (37% and 23%, respectively), while those with lower connectivity such as hydrolases and other enzymes had a much lower recovery (1% and 0.7%, respectively). In the case of the MetaBase™ network, we found that nuclear receptors were recovered frequently (22%) despite their relatively low connectivity compared to protein kinases and other cytosolic proteins. This could be due to the fact that nuclear receptors are just upstream from transcription factors [58] hence such targets are recovered more easily from transcriptomics data. The findings were consistent with the observed correlations in Fig. 5, in that the protein class recovery was more dictated by node connectivity with the Omnipath network compared to the MetaBase™ network.

**Fig. 7:**
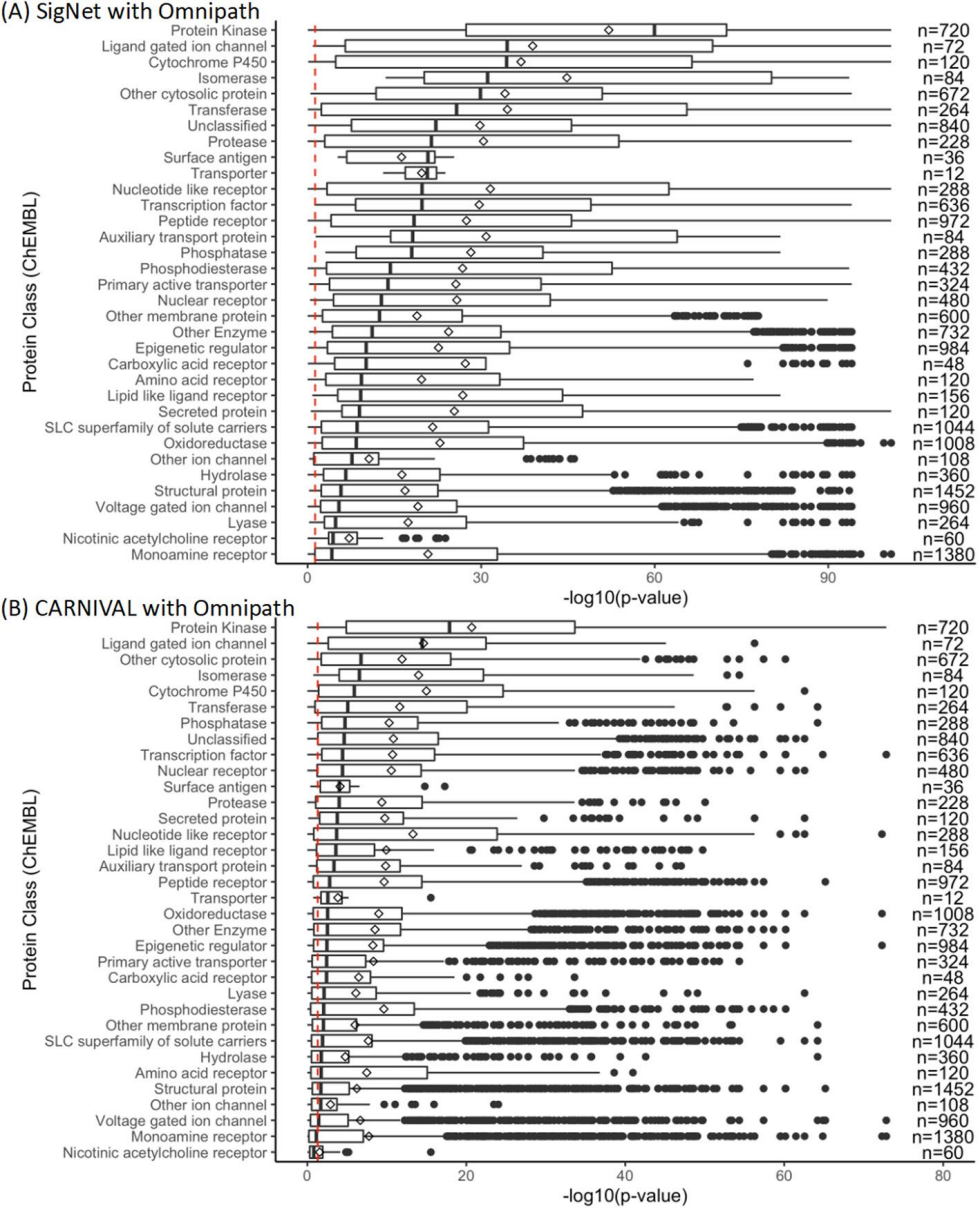
Distributions of the significance of compound-associated pathway enrichment based on causal nodes from **A** SigNet and Omnipath and **B** CARNIVAL and Omnipath, across all combinations of factors, separated into the protein classes targeted by each compound. Mean values are indicated by a diamond shape in the box plot. The number of instances is annotated on the right-hand side of the plot. The significance threshold p = 0.05 is indicated with a red dashed line

We next sought to understand how the recovery of signalling pathways related to protein class and function. To this end, we plotted the distribution of compound target-associated pathway enrichment significance for compounds targeting proteins in different classes (Fig. 7). We chose to focus on SigNet and CARNIVAL with Omnipath because both showed a high performance, SigNet in terms of statistical significance, and CARNIVAL in terms of recovering informative pathways. In general, we found the highest performance for compounds targeting protein kinases and ligand-gated ion channels, which are both key mediators of cellular signalling—protein kinases transmit cellular signals through phosphorylation, and ligand-gated ion channels function to receive and transmit signals. The worst performance was seen for compounds targeting nicotinic acetylcholine and monoamine receptors; these receptors modulate signalling in the CNS [59, 60], and were additionally not expressed in high levels in the breast-cancer and prostate-cancer cell lines used (Additional file 1: Fig. 5) hence we propose that the biological context of the cell-lines used influenced the results. We hypothesise that gene expression data measured in biological models derived from the CNS would lead to a higher recovery of such signalling pathways. We note that this particular analysis was complicated by the fact that compounds can target proteins from multiple classes.

Overall, the results of this section show that the performance of Causal Reasoning algorithms for recovering compound targets, and compound-associated pathways, is not equal across protein classes. The connectivity of targets on the prior knowledge network were shown to heavily impact their direct recovery by the algorithms, with algorithms which correct for uneven connectivity (CausalR) showing less of an association between target degree and its successful recovery. The biological role of the considered targets was also reflected in the results, with protein kinases recovered most successfully both in terms of direct target recovery, and the recovery of relevant pathways. A potential way to mitigate the connectivity bias is for random simulation studies be carried out to identify which network nodes may be recovered by chance, an approach which has been used previously [61]. We note that one argument against this is that well-connected nodes in networks are well-studied, and have found to be essential proteins with key roles in diseases [56], and correcting against them could lead to discarding potential true positives, so that the bias described in this section is at least to an extent also desired and useful for elucidating compound targets.

### Case study: factors affecting MoA recovery of Diethylstilbestrol

**Fig. 8:**
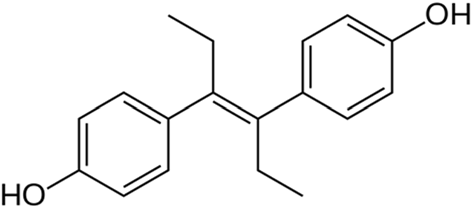
Structure of diethylstilbestrol, a non-steroidal estrogen drug included in the benchmarking study

To contextualise our findings, we present a case study investigating the factors affecting target and pathway recovery of the drug Diethylstilbestrol (Fig. 8) which was approved to treat prostate and breast cancer. This compound was part of the set used to benchmark this study, and the 33 targets retrieved for this compound included its canonical targets (estrogen receptors ESR1 and ESR2) as well as its other protein targets measured with in vitro assays retrieved from the ChEMBL database (as described in Materials and Methods). The full list of targets in HGNC symbols can be found along with the full benchmarking results for Diethylstilbestrol across all combinations of parameters in Additional file 4.

Table 5 shows, for each parameter, the number of correctly retrieved annotated targets for each level averaged across all other parameter levels. It can be seen that the parameter with greatest influence on target recovery is the algorithm with a range of 3.56. SigNet recovers the highest average number of targets (4.40) while CARNIVAL recovers the lowest average number of targets (0.83), which matches the overall trends summarised in Fig. 2.

**Table 5:** Target recovery (direct target overlap with causal reasoning output nodes) averaged for each parameter level for the benchmark results for compound Diethylstilbestrol

Platform		Gene Set		Cell Line		Algorithm		Network	
LINCS	2.24	ALL	2.30	MCF7	2.32	CausalR (Results table)	1.54	Omnipath	1.75
CMap	2.33	LM	1.97	PC3	2.25	CARNIVAL	0.83	Metabase All	2.00
		BING	2.59			CausalR (Network)	2.38	Metabase High	2.69
						SigNet	4.40	Metabase Med	2.71
Range	**0.09**		**0.63**		**0.07**		**3.56**		**0.96**

The range across parameter levels is given in bold

In terms of canonical targets only, across all 48 potential combinations of platform, gene set, cell line and network, SigNet was able to recover ESR1 every time, and ESR2 in all cases except when using the Omnipath network (as ESR2 was not included in this network). Despite CARNIVAL recovering the lowest average number of targets, a canonical target was recovered across 20 combinations of the other parameters, or in around 42% of cases. On the other hand, the CausalR results table, which recovered a higher average number of all targets, only recovered a canonical target in 10 cases (~ 21%). Finally, CausalR ScanR network recovered a canonical target in 31 cases (~ 65%) (Results shown in Additional file 4). This supports our overall finding that SigNet is the best algorithm for recovering compound targets using causal reasoning.

Table 6 shows, for each parameter, the two-step pathway enrichment score for each level averaged across all other parameter levels. In agreement with our overall findings, it can be seen that by far the parameters with greatest influence on pathway recovery were the choice of network (range of 16.2) and algorithm (range of 41.6) where SigNet was the best performing algorithm and Omnipath the best performing network.

**Table 6:** Pathway recovery (over-representation of enriched pathways from causal reasoning nodes in the set of compound target-associated pathways expressed as a − log10(p-value)) averaged for each parameter level for the benchmark results for compound Diethylstilbestrol

Platform		Gene Set		Cell Line		Algorithm		Network	
LINCS	26.5	ALL	24.3	MCF7	24.9	CausalR (Results table)	4.33	Omnipath	36.7
CMap	23.8	LM	27.0	PC3	25.5	CARNIVAL	15.9	Metabase_All	21.5
		BING	24.2			CausalR (Network)	34.6	Metabase_High	20.6
						SigNet	46.0	Metabase_Med	22.0
Range	**2.70**		**2.77**		**0.59**		**41.6**		**16.2**

The range across parameter levels is given in bold

In terms of canonical pathways related to the primary mechanism of action of Diethylstilbestrol, we counted the number of cases across different parameter combinations where recovered pathways contained the text “estrogen” or “ESR” (e.g., “ESR-mediated signalling” or “Extra-nuclear estrogen signalling”). These are summarised in Table 7 (full results are in Additional file 4) and show that unsurprisingly (as ESR1 or ESR2 were directly recovered in every case as discussed above), SigNet recovered estrogen-related pathways across all other parameter combinations.

**Table 7:** Number of cases across different parameter combinations where the enriched pathways contained text “ESR” or “Estrogen” (canonical mechanistic pathways) for Diethylstilbestrol

Algorithm	Cases (% out of 48)	Cases when Network = Omnipath (% out of 12)
CausalR (Results table)	10 (20.8%)	3 (25.0%)
CARNIVAL	38 (79.2%)	11 (91.7%)
CausalR (Network)	45 (93.8%)	10 (83.8%)
SigNet	48 (100%)	12 (100%)

Considering all possible parameter combinations using the CARNIVAL algorithm, 38 out of 48 or 79.2% of cases recovered estrogen-related pathways, while when just considering the Omnipath network results, 11 out of 12 or 91.7% of cases recovered estrogen-related pathways. This agrees with the overall trends shown in Fig. 5, which suggest that many more compound-associated pathways are recovered when using the Omnipath network with CARNIVAL compared to the larger MetaBase™ networks. With the exception of the CausalR results table, the algorithms showed a good ability to recover estrogen-related pathways across parameter combinations, in particular with the Omnipath network (91.7%, 83.8% and 100% of cases), despite the direct targets themselves being recovered in fewer cases. These results (put together with the overall findings of the benchmarking study) indicate that, to obtain the best chance of understanding the mechanism of action of a compound with causal reasoning, the output nodes of the algorithms should be used to perform pathway enrichment analysis.

### Limitations of this study

While we aimed for a comprehensive parameter exploration and benchmarking of causal reasoning algorithms in this work, it still has some limitations as well. Firstly, we were limited by the annotations available for the compounds used, and chemical space (and mode of action) coverage in this set in the first place. This has profound implications for our work (and indeed, in any work where a ‘ground truth’ must be set for the mode of action of compounds): Different areas of chemical and mode of action space behave differently, and assuming unavailable data as inactive punishes ‘false positives’, which may very well be novel true positives which are just not annotated as such.

The mechanisms of action under investigation in this study were specifically small molecules with human protein target-mediated mechanisms, but not all compounds achieve their desired pharmacological effects in this way. For example, some anti-bacterial agents act by inhibiting cell wall synthesis in bacterial cells. Such mechanisms of action would not be identified using the approach undertaken in this study.

We were additionally not able to benchmark all available causal reasoning algorithms due to the high computational cost of the algorithms which scale with network size. Further benchmarking should be carried out for other causal reasoning algorithms. Furthermore, the causal reasoning algorithms additionally infer node directionality (i.e., whether the recovered signalling proteins activated or inhibited), which we did not consider when benchmarking the results as it was not possible to obtain consistent and complete functional pharmacology information about the compound-target interactions (i.e., are they activated or inhibited upon pharmacological modulation).

Furthermore, the cell lines used in the gene expression experiments considered (MCF7 and PC3) are quite similar in terms of baseline gene expression (Additional file 1: Fig. 5) which is why we potentially did not see any significant difference in performance when using data derived from either cell line, but these are the only cell lines with data available on a large scale in the original CMap. Previous studies using other methodologies have shown that relevant transcriptional signals can be found in cell lines which are seemingly physiologically irrelevant; for example, machine learning models to predict drug-induced liver injury (DILI) were trained on transcriptional profiles derived from MCF7 and PC3 cell lines, and important features (genes) were involved in liver metabolism pathways [62]. Another DILI study found that transcriptional signatures in HL60 (leukaemia) cells had a high similarity (Pair Rank score 0.70) to those measured in human primary hepatocytes “suggesting the two assays could be potentially interchangeable” [63], and a drug repurposing study was able to elucidate CNS receptor targets for a small molecule using LINCS cancer cell line data [64]. Although we found in our study that the mechanisms of action of compounds targeting CNS-related receptors (e.g., monoamine receptors) were not recovered on the pathway-level as well as those targeting protein kinases, transcription factors and other receptors more relevant to the cancer cell lines used (Fig. 7), we were not able to systematically evaluate the applicability of causal reasoning to recover diverse mechanisms using data derived from a wide range of cell lines. As LINCS provides data derived from 99 cell types, a future extension to this study could be to assess the performance of causal reasoning methods using data derived from other cell lines and relate these to the applicability domain of the methodologies—for example, non-cancer cell lines from a variety of tissues such as HA1E (normal kidney) or CNS cells such as NPC/NEU, and quantify to what extent cell lines can be used interchangeably when using this approach to elucidate mechanisms of action. It is also important to note that cell lines are in vitro models which cannot necessarily recapitulate in vivo processes, and the limitations of using cell lines in general has been extensively discussed [65, 66].

## Conclusions and recommendations

In this study we performed a comprehensive benchmarking of the SigNet, CausalR and CARNIVAL causal reasoning algorithms to recover compound mechanism of action from L1000 and CMap transcriptomics data, measured in the MCF7 and PC3 cell lines, and using four different prior knowledge networks. By considering two evaluation metrics, on the direct target level and the pathway level, and using statistically modelling, we were able to identify the factors which had the most significant influence on MoA recovery. This conclusion section will summarise the key findings and also use them to provide guidelines for researchers wanting to implement these causal reasoning algorithms.

In terms of the performance of recovering direct targets, we found that the choice of network and algorithm were qualitatively the most significant factors, indicating that networks behave rather differently with different prior networks. In particular, the larger MetaBase™ networks were found to be more suitable with the CausalR scoring function to discover direct compound targets—while CARNIVAL and SigNet performed better with the smaller Omnipath network. SigNet with the Omnipath network achieved the highest performance for direct target recovery. The other individual factors and interaction effects, namely the choice of platform (LINCS L1000 or CMap microarray), cell line (MCF7 or PC3), and gene set (landmark, landmark and best inferred, or all genes) were either not found to be statistically significant or did not result in any practically relevant changes in performance. These results indicate that the LINCS L1000 data is suitable for use with causal reasoning algorithms, at least based on the other factors used in this work, presenting an opportunity for novel research to be carried out using this large dataset. They also indicate that the 978 landmark genes are truly informative enough to gain insight into compound mechanism of action, as hypothesised in the original publication [7]. For recovering direct targets, we hence recommend that the SigNet algorithm with the Omnipath network should be used for the best performance, and can also be used with data derived from either microarray or L1000 transcriptomics platform.

We also examined the average performance for each combination of network and algorithm when recovering compound-associated pathways, and quantified how specific (and therefore informative for MoA understanding) the recovered pathways were, in terms of their position in the Reactome hierarchy and their gene set size. CARNIVAL, though less appropriate for discovering direct targets, was able to recapitulate networks representative of specific and informative pathways encompassing actual compound targets. The CausalR ranked table showed the worst performance, while SigNet again showed the best performance with the Omnipath network. Notably, we found that the causal reasoning-derived nodes generally outperformed differentially expressed genes (DEGs) as input for pathway enrichment both in terms of the statistical significance of enrichment in compound-derived pathways *and* how informative the pathways were. Based on these results we recommend that performing causal reasoning using SigNet, CARNIVAL or ScanR could be a better strategy for understanding modulated signalling pathways compared to traditional pathway enrichment on the transcript-level. Our case study with the compound Diethylstilbestrol along with the overall results also indicate that in general, the algorithms are better utilised for recovering modulated pathways rather than the direct target(s) of compounds.

Furthermore, we sought to understand the applicability domain of the methods with regards to target function and network biases. We found that target recovery heavily depends on target connectivity (number of edges) in a given network, and that the CausalR ranked table (which corrects for degree bias) had the lowest concordance between connectivity and recovery. This has two effects: On the one hand, this penalises hub nodes, which often represent disease targets (52) (and this lack of bias may hence also be related to the relatively low performance in terms of direct target recovery). On the other hand, for atypical (or incompletely annotated targets) this type of behaviour may be rather beneficial. Furthermore, we found that the biological role of the proteins influenced their successful recovery: transcription factors and nuclear receptors were most often recovered as direct targets, and protein kinases and mediators of cellular signalling on the pathway level. We also found that CNS-related pathways were not often recovered, corresponding to their low baseline expression in the MCF7 and PC3 cell lines used to derive transcriptomics data. It is hence recommended to choose an in vitro cell line that is best able to recapitulate in vivo disease/phenotypes—for example, breast cancer cell lines which have been found to have genomic similarity to breast cancer patient samples [67]. To correct for hub node bias, it is recommended to carry out random simulation studies to identify which network nodes may be recovered by chance [61].

Overall, we were in this work hence able to explore performance, and for the first time other factors that influence performance (prior knowledge network, biological properties and annotations of the target/pathway, input transcript data), when using gene expression data in combination with causal reasoning algorithms for mechanism of action analysis, which provides guidelines for their use by researchers in this field in the future.

## Supplementary Information

**Additional file 1**: Supplementary figures and tables

**Additional file 2:** Supplementary methods.

**Additional file 3:** List of compounds considered in the benchmarking study with extracted bioactivity information.

**Additional file 4:** Results for the case study Diethylstilbestrol.

## Data Availability

The transcriptomics datasets used in this study are available in the GEO (https://www.ncbi.nlm.nih.gov/geo), accession numbers *GSE92742* and *GSE70138* and https://portals.broadinstitute.org/cmap. The Omnipath network used in this study is available at https://omnipathdb.org/. The MetaBase™ networks used in this study are available under a license from Clarivate™. You may not copy or re-distribute this material in whole or in part without the written consent of Clarivate™.

## Abbreviations

MoA: Mechanism of action
DEG(s): Differentially expressed gene(s)
CMap: Connectivity map
PPI: Protein–protein interaction
PKN: Prior knowledge network
LM: Landmark
TF: Transcription factor
