# Supplementary material for: Benchmarking causal reasoning algorithms for gene expression-based compound mechanism of action analysis
Source: BMC Bioinformatics. 2023 Apr 18;24:154. doi: 10.1186/s12859-023-05277-1 (PMC10111792; doi:10.1186/s12859-023-05277-1)
Supplement: Supplementary file 1 — Additional file 1: Supplementary figures and tables [file 12859_2023_5277_MOESM1_ESM.docx]

**Supplementary Information**

Supplementary Figures


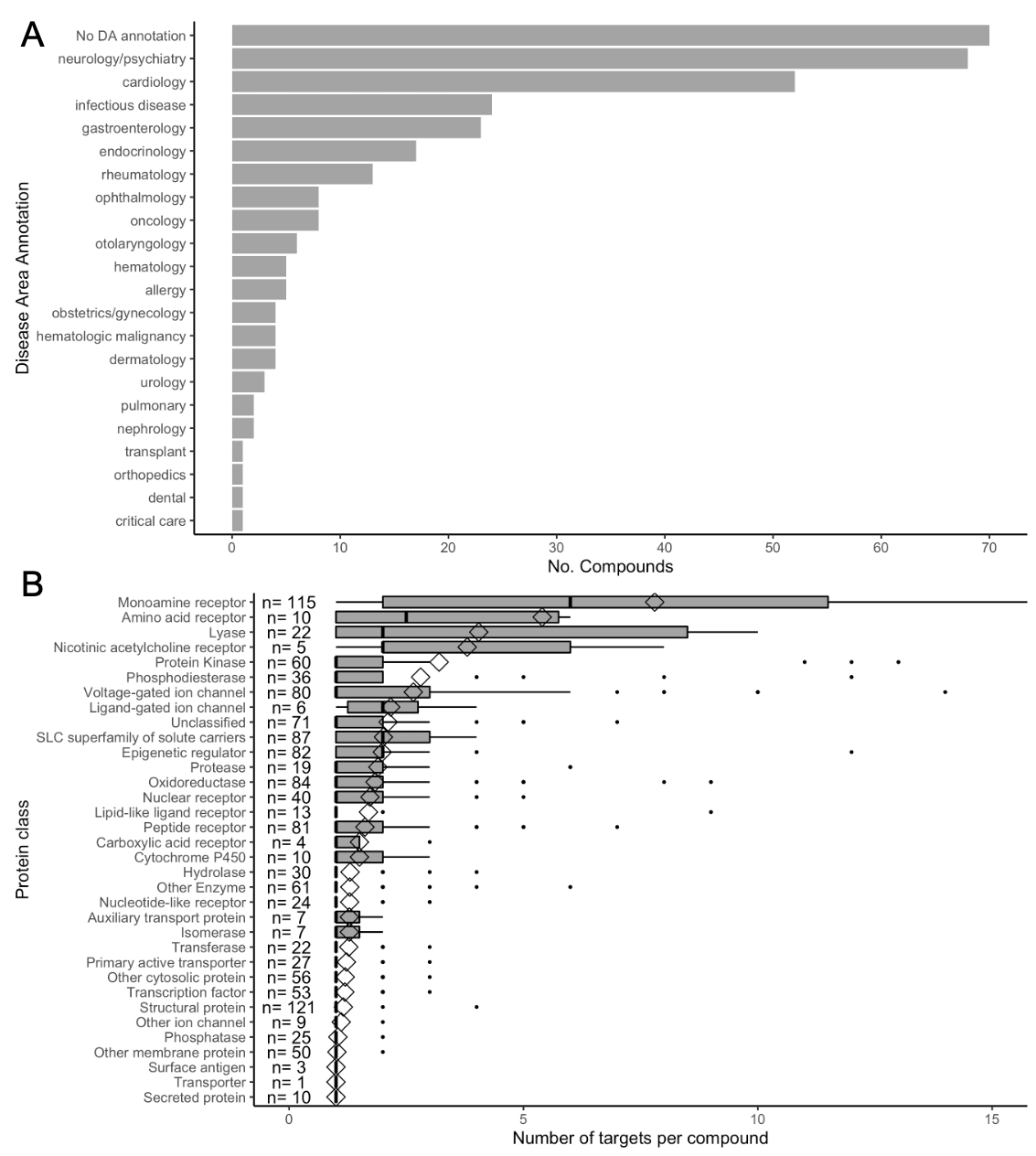


Supplementary Figure 1: Distribution of Disease Area annotations (A) and target protein classes (obtained from ChEMBL) (B) in the set of compounds used in this study. The number of unique compounds targeting proteins in each class is annotated next to each protein class label.


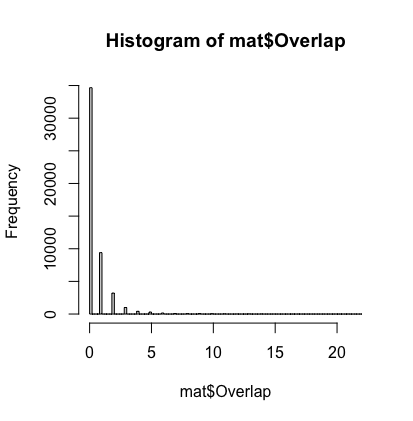


Supplementary Figure 2: Poisson distribution of “Overlap” (direct target recovery) evaluation metric modelled by Type II Negative Binomial model


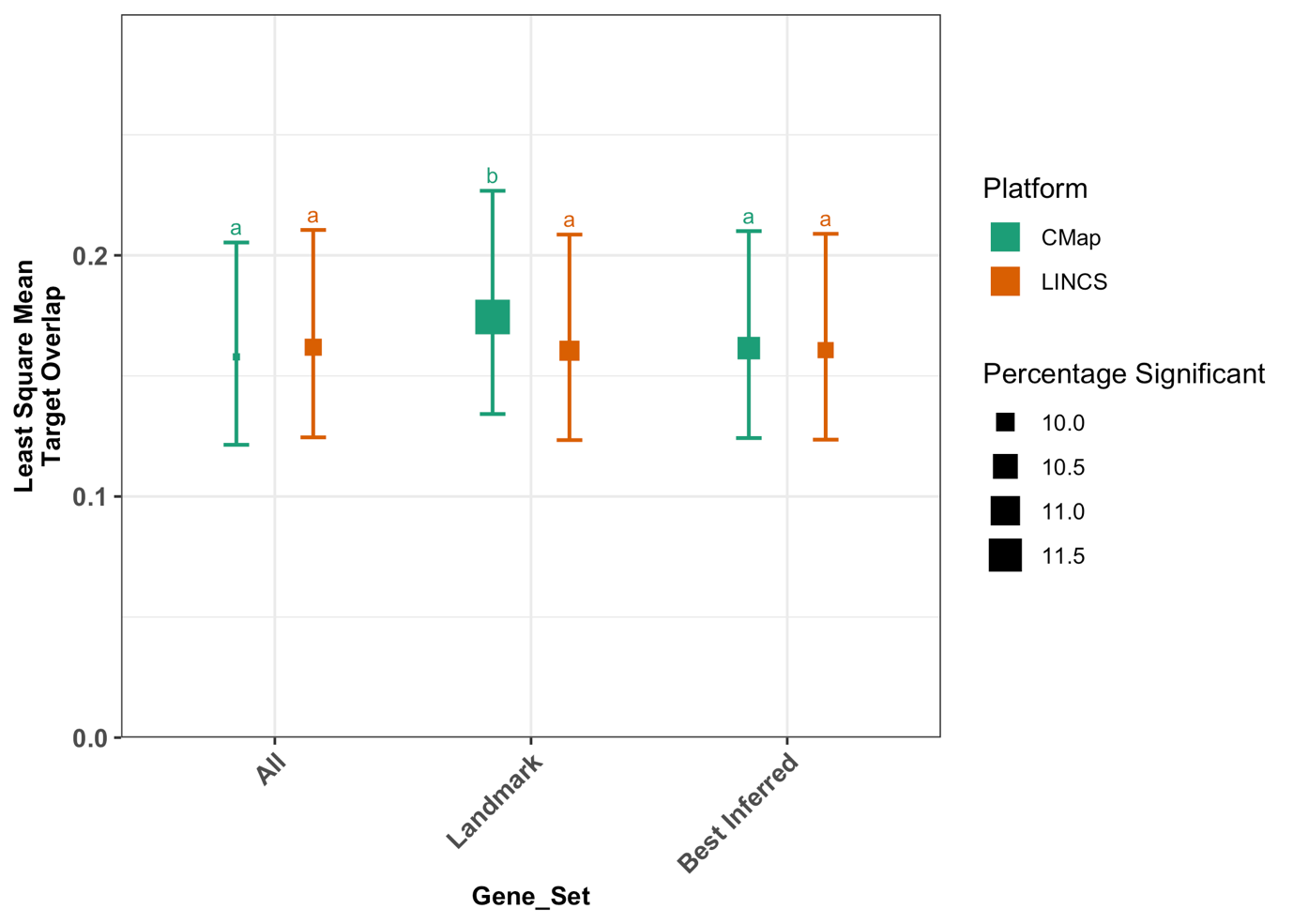


Supplementary Figure 3: Interaction plot showing post-hoc least square means across all other factors for the Platform:Gene Set interaction effect from the negative binomial model of the target recovery evaluation metric. Means sharing a letter are not significantly different according to pairwise comparisons of least square means, with Sidak adjusted p-values for multiple comparisons. Error bars indicate the least square means Sidak 95% confidence interval. It can be seen here that the confidence intervals overlap and hence this interaction effect was not studied any further.


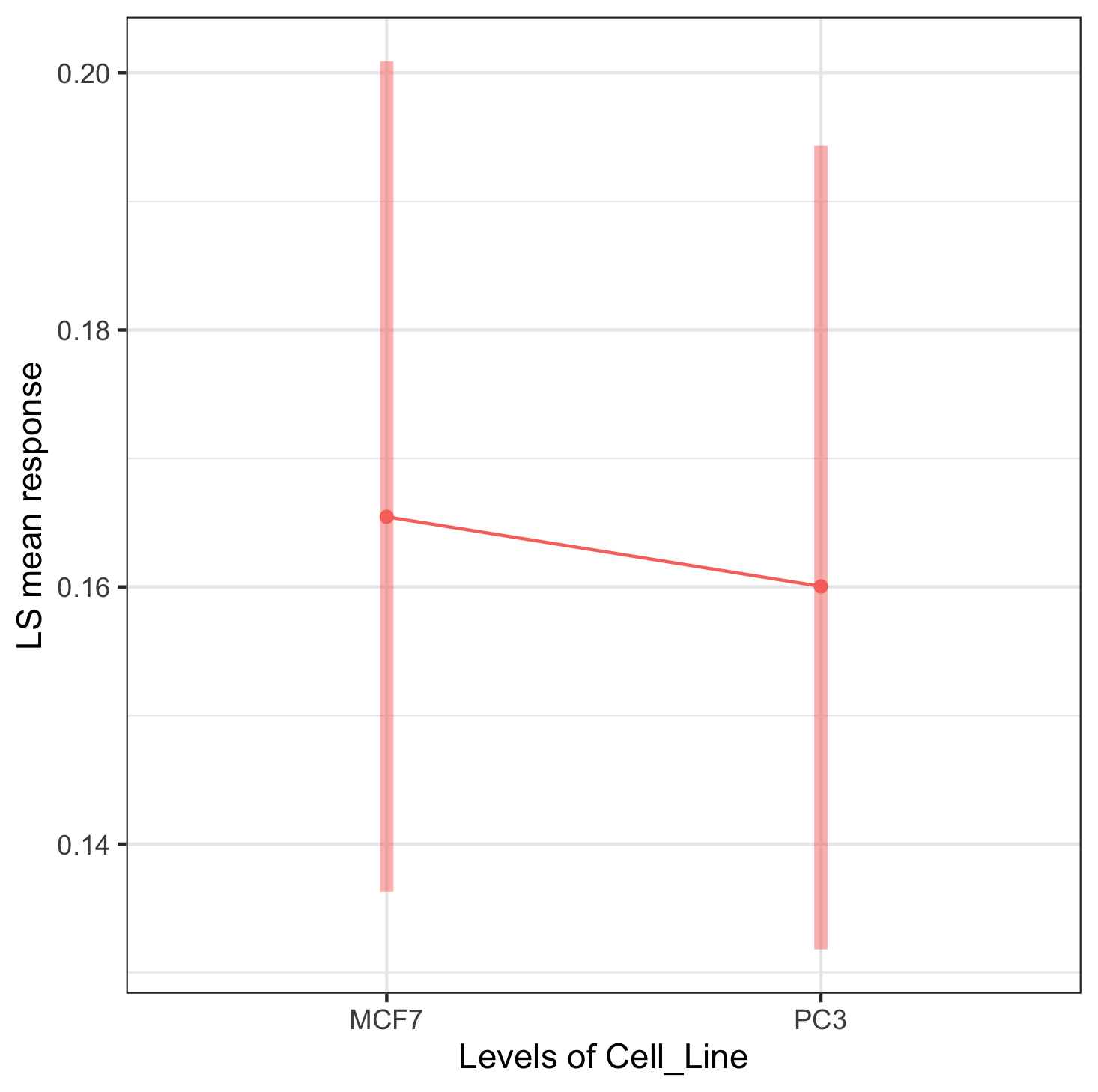


Supplementary Figure 4: Least-square mean response and 95% confidence intervals for the Cell Line effect from the negative binomial model of the target recovery evaluation metric. It can be seen here that the confidence intervals overlap and hence this effect was not studied any further.


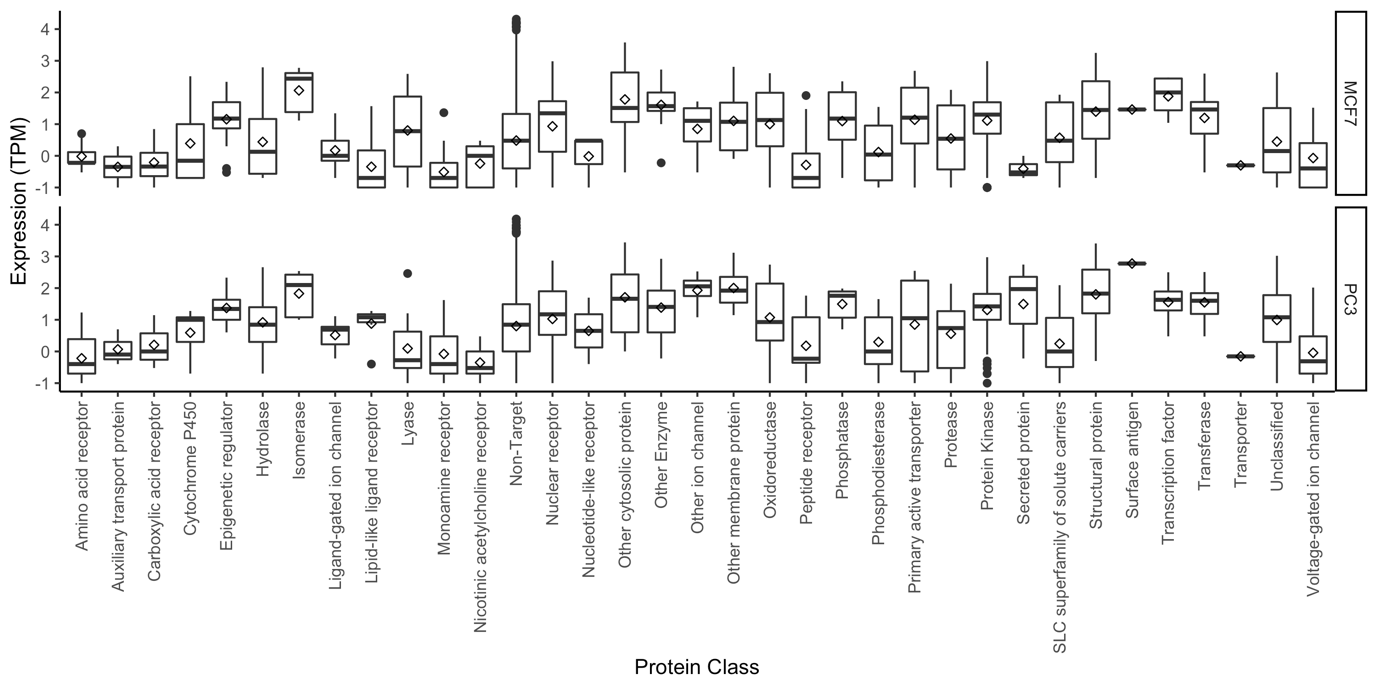


Supplementary Figure 5: RNA-Seq expression level (log10 TPM or Transcripts Per Million) distribution per-protein class (ChEMBL), baseline expression in MCF7 and PC3 cell lines, data from ExpressionAtlas^1^ .

Supplementary Figure 6: Distributions of the degree of connectivity for target vs. non-target nodes on each network**.** Each distribution was compared with a one-sided Wilcoxon rank sum test to assess the probability of the target nodes being more densely connected than non-target nodes by random chance, found to be significant (p < 0.0001) in all cases.

Supplementary Tables

Supplementary Table 1: Statistics relating to the 4 prior knowledge networks used in this analysis, including number of nodes and edges, network density (median and mean degree of nodes), and their coverage of the targets modulated by the compounds in the test set

| **Network** | **Nodes** | **Edges** | **Median Degree** | **Mean Degree** | **Target Coverage**  **N = 681** |
| --- | --- | --- | --- | --- | --- |
| Omnipath | 3610 | 9306 | 2 | 5 | 49.5% (337) |
| Metabase  (All confidence levels) | 11004 | 87556 | 5 | 16 | 85.7% (584) |
| Metabase  (Medium confidence) | 9523 | 57758 | 4 | 12 | 79.7% (543) |
| Metabase  (High confidence) | 9148 | 51730 | 4 | 11 | 78.4% (534) |

Supplementary Table 2: Negative binomial model parameters, estimates and probabilities for the target overlap metric

|  | **Estimate** | **Std. Error** | **z value** | **Pr(>\|z\|)** |
| --- | --- | --- | --- | --- |
| (Intercept) | -3.56169 | 0.12076 | -29.49 | < 2e-16 |
| NetworkMetabase_High | 0.56869 | 0.08468 | 6.72 | 1.87E-11 |
| NetworkMetabase_Med | 0.5397 | 0.08511 | 6.34 | 2.27E-10 |
| NetworkOmnipath | 1.63983 | 0.07426 | 22.08 | < 2e-16 |
| AlgorithmCausalR_Network | 2.19909 | 0.07175 | 30.65 | < 2e-16 |
| AlgorithmCausalR_RT | 2.06618 | 0.07228 | 28.59 | < 2e-16 |
| AlgorithmSigNet | 2.48531 | 0.07087 | 35.07 | < 2e-16 |
| PlatformLINCS | 0.02508 | 0.02172 | 1.15 | 0.248235 |
| Gene_SetBING | 0.02274 | 0.02173 | 1.05 | 0.295338 |
| Gene_SetLM | 0.09961 | 0.02135 | 4.67 | 3.06E-06 |
| Cell_LinePC3 | -0.03331 | 0.01243 | -2.68 | 0.007366 |
| NetworkMetabase_High:AlgorithmCausalR_Network | -0.65612 | 0.09058 | -7.24 | 4.38E-13 |
| NetworkMetabase_Med:AlgorithmCausalR_Network | -0.64124 | 0.09101 | -7.05 | 1.85E-12 |
| NetworkOmnipath:AlgorithmCausalR_Network | -2.38293 | 0.08404 | -28.36 | < 2e-16 |
| NetworkMetabase_High:AlgorithmCausalR_RT | -0.65574 | 0.09137 | -7.18 | 7.15E-13 |
| NetworkMetabase_Med:AlgorithmCausalR_RT | -0.51799 | 0.09142 | -5.67 | 1.46E-08 |
| NetworkOmnipath:AlgorithmCausalR_RT | -2.93466 | 0.09027 | -32.51 | < 2e-16 |
| NetworkMetabase_High:AlgorithmSigNet | -0.48859 | 0.08881 | -5.5 | 3.77E-08 |
| NetworkMetabase_Med:AlgorithmSigNet | -0.4708 | 0.08923 | -5.28 | 1.32E-07 |
| NetworkOmnipath:AlgorithmSigNet | -1.52635 | 0.07902 | -19.32 | < 2e-16 |
| PlatformLINCS: Gene_SetBING | -0.03037 | 0.03066 | -0.99 | 0.321868 |
| PlatformLINCS:Gene_SetLM | -0.10874 | 0.03039 | -3.58 | 0.000346 |

Supplementary Table 3: CLD (Compact Letter Display) results for the Network:Algorithm interaction effect showing the least-square mean response, 95% confidence intervals, and their CLD group (where means sharing a letter are not significantly different). The % signif column shows the % of cases where the number of targets recovered for a compound was statistically significant (p <= 0.05) according to Fisher’s Exact Test when accounting for the number of targets, output nodes and network nodes.

| Network | Algorithm | response | lower.CL | upper.CL | group | % signif |
| --- | --- | --- | --- | --- | --- | --- |
| Metabase_All | CARNIVAL | 0.0288 | 0.0202 | 0.041 | a | 3.21 |
| Metabase_Med | CARNIVAL | 0.0494 | 0.0356 | 0.0684 | b | 3.7 |
| Metabase_High | CARNIVAL | 0.0508 | 0.0367 | 0.0704 | b | 3.4 |
| Omnipath | CausalR_RT | 0.0622 | 0.0452 | 0.0857 | b | 1.27 |
| Omnipath | CausalR_Network | 0.1234 | 0.0909 | 0.1675 | c | 7.76 |
| Omnipath | CARNIVAL | 0.1483 | 0.1096 | 0.2008 | d | 6.14 |
| Metabase_High | CausalR_RT | 0.2082 | 0.1544 | 0.2809 | e | 3.43 |
| Metabase_All | CausalR_RT | 0.2272 | 0.1685 | 0.3062 | ef | 3.77 |
| Metabase_Med | CausalR_RT | 0.2322 | 0.1723 | 0.3129 | efg | 4.37 |
| Metabase_Med | CausalR_Network | 0.2344 | 0.174 | 0.3159 | efg | 14.25 |
| Metabase_High | CausalR_Network | 0.2377 | 0.1765 | 0.3203 | fg | 14.47 |
| Metabase_All | CausalR_Network | 0.2595 | 0.1927 | 0.3494 | g | 14.47 |
| Metabase_All | SigNet | 0.3455 | 0.257 | 0.4643 | h | 20.93 |
| Metabase_Med | SigNet | 0.3701 | 0.2754 | 0.4973 | hi | 21.57 |
| Metabase_High | SigNet | 0.3743 | 0.2786 | 0.5028 | hi | 22.19 |
| Omnipath | SigNet | 0.387 | 0.2878 | 0.5203 | i | 23.36 |

References:

1 I. Papatheodorou, N. A. Fonseca, M. Keays, Y. A. Tang, E. Barrera, W. Bazant, M. Burke, A. Füllgrabe, A. M.-P. Fuentes, N. George, L. Huerta, S. Koskinen, S. Mohammed, M. Geniza, J. Preece, P. Jaiswal, A. F. Jarnuczak, W. Huber, O. Stegle, J. A. Vizcaino, A. Brazma and R. Petryszak, Expression Atlas: gene and protein expression across multiple studies and organisms, *Nucleic Acids Res*, 2018, **46**, D246–D251.
