# Supplementary material for: Benchmarking causal reasoning algorithms for gene expression-based compound mechanism of action analysis
Source: BMC Bioinformatics. 2023 Apr 18;24:154. doi: 10.1186/s12859-023-05277-1 (PMC10111792; doi:10.1186/s12859-023-05277-1)
Supplement: Supplementary file 2 — Additional file 2: Supplementary methods. [file 12859_2023_5277_MOESM2_ESM.docx]

**Supplementary Methods**

1. **Data extraction**

For this study, we extracted compound signatures measured in the PC3 and MC7 cell lines at 6h and 10µM. L1000 data (Level 5 – differential gene expression signatures) [1]were extracted (access date September 2019) using the CMapPy[2] package from .gctx files found at GEO accessions *GSE92742* (Phase I) and *GSE70138* (Phase II), facilitated with the use of a Python script available on GitHub (<https://github.com/BenderGroup/LINCS-Extraction>). The Python script uses the metadata files, also available at the GEO accessions, to query the L1000 data meeting specified conditions (cell line, time-point, dose). Following data extraction, the script collapses “biological replicate” signatures into a consensus signature by weighting each replicate based on co-correlation (as described for “technical replicates” in the L1000 protocol[1]). CMap data was extracted using the protocol and functions described in longevityTools R package eDRUG vignette[3], which included the extraction of CEL files from the CMap website (https://portals.broadinstitute.org/cmap/), normalisation with MAS5[4] and differential analysis with *limma*[5]. This produced a total of 2,016 unique compounds measured in both data sets under the specified conditions. To investigate the effect of including inferred transcriptomics data on mechanism of action recovery, both the LINCS and CMap data were separated into three subsets – landmark genes only (based on the definition of landmark genes from the L1000 study found in the “gene_info.txt” metadata), landmark and “best inferred genes” (again based on the L1000 metadata), and landmark and all inferred genes.

1. **Target Annotation**

For each of the 2,016 compounds, LINCS “pert_info.txt” metadata files were used to map compound preferred name (pert_iname) to InChI key. InChI keys for compounds were queried *via* the ChEMBL 25[6] mySQL database, with any protein bioactivity measurement corresponding to an activity of 10µM (pChEMBL of >=5) or better being annotated to the compound as a target. Target annotations for each compound were also extracted from the clue.io Repurposing Hub (2019)’s downloadable .CSV file. Both target lists were harmonized for each compound to produce a list of unique target annotations as HGNC symbols, and compounds with no target annotations were dropped. This formed a set of 269 compounds with bioactivity data and transcriptomic data measured in both LINCS and CMap under the specified conditions (Additional File 1 shows the list of compounds and their targets). Disease Area annotations were also obtained from clue.io Repurposing Hub for the 269 compounds, where available. Targets were mapped to their corresponding protein classes using the ChEMBL webresource client Python package (v. 0.10.0).

1. **Prior Knowledge Networks**

**Omnipath**

The Omnipath[7] protein-protein interaction (PPI) network (signed and directed edges only) was extracted from the CARNIVAL[8] supplementary data with nodes as UniProt IDs. The IDs were converted to HGNC symbols using the *org.Hs.eg.db* package (v. 3.8.2)[9]. For SigNet, additional transcriptional regulatory interactions were extracted from OmnipathDB using the ‘tf_target’ and ‘dorothea’ filters, and concatenated with the PPI network (accessed November 2019).

**MetaBase**

The entire MetaBase™ network was first extracted using the get.globalnetwork(species=”human”) function of metabaseR (v. 4.2.3, November 2019, Thomspon Reuters^TM^). Edges that were not direct molecular interactions were removed. Network object IDs were then converted to their corresponding Entrez IDs using the CBDD (November 2019, Thompson Reuters^TM^) convertNetworkObjects2Entrez(networkobject,species=”human”) function. Nodes which did not convert to Entrez IDs were removed. If one network object corresponded to multiple Entrez IDs – i.e. a protein complex or a family – it was assumed that all members of the protein complex take part in the interaction. Only signed and directed edges were kept. To retain only protein-protein interactions from the global network, interactions annotated with “co-regulation of transcription”, “Transcription regulation”, “Influence on expression”, “miRNA binding”, “Unspecified”, or “Pharmacological effect” were removed. For SigNet, to retain protein-protein and transcriptional regulatory interactions, only the interactions with mechanism “miRNA binding”, “Unspecified” and “Pharmacological effect” were removed. Each network was then split into three confidence levels based on the “trust” attribute of each interaction (low, medium, high) – high confidence interactions only, high and medium interactions, and all interactions including low, medium and high confidence.

1. **Causal Reasoning Pipeline**

**TF enrichment with DoRothEA and pathway scoring with PROGENy**

For CARNIVAL and CausalR, signatures were processed further to yield transcription factor (TF) activities (this step was not necessary for SigNet as the algorithm uses transcriptional regulation interactions in the prior knowledge network to link gene expression data to TF activities as the first step). For the CMap data, genes were input with their log2 fold-change values into DoRothEA, which uses a consensus TF-gene regulon to compute enrichment scores for transcription factors[10]. For the LINCS data, gene Z-scores were input into DoRothEA.

As additional input to CARNIVAL, the same process was repeated with the PROGENy pipeline[11] to obtain pathway scores derived from a common core of pathway responsive genes, generated from a large compendium of perturbation experiments

**Causal Reasoning**

For this benchmarking study we used three different R packages, SigNet[12] (implemented in CBDD) CausalR[13] and CARNIVAL[8].

CausalR (v. 1.16.0) was run with DoRothEA TF activities with the ReadExperimentalData() function. PPI networks were converted to computational causal graphs using the CreateCCG() function with default parameters. Two different outputs were obtained for this study. The “Ranked Table” output was obtained by running RankTheHypotheses(delta=5) where 5 denotes the maximum number of path lengths traversed. To generate a subnetwork output, the ScanR() functionality was modified slightly and the R code can be found at https://github.com/laylagerami/CausalReasoningBenchmark/. The original function returns the top N ranked regulators across a user-defined level of path lengths, whereas the modified function instead returns the regulators with a significant (p <= 0.05) score. The method returns “consensus regulators” across the maximum number of path lengths traversed, from which a subnetwork is formed by connecting these to the input TFs *via* correctly scored (concordant) interactions.

The CARNIVAL (v. 0.9.3) *InvCarnival* algorithm (where known targets are not supplied as input) was run on the TF activities from DoRothEA and pathway scores from PROGENy using the runCARNIVAL() function with a time limit of 900 seconds and all other parameters kept as their default. The OmniPath and MetaBase™ PPIs were used as prior knowledge networks. The final pooled consensus subnetwork was taken as output.

The SigNet algorithm was applied to all datasets with the Log2FC (CMap) or Z-score (LINCS) used as input using the PPI networks with the additional transcriptional regulation interactions. The algorithm was run using the SigNet() function in the *CBDD* package. All parameters were kept as default (path length of 5), and the ranked table was used as output. Directionality was not considered in this study, and ranked tables were concatenated so as to only consist of the highest ranked sign of each protein.

1. Subramanian A, Narayan R, Corsello SM, Peck DD, Natoli TE, Lu X, et al. A Next Generation Connectivity Map: L1000 Platform and the First 1,000,000 Profiles. Cell. 2017;171:1437-1452.e17.

2. Enache OM, Lahr DL, Natoli TE, Litichevskiy L, Wadden D, Flynn C, et al. The GCTx format and cmap{Py, R, M, J} packages: resources for optimized storage and integrated traversal of annotated dense matrices. Bioinforma Oxf Engl. 2019;35:1427–9.

3. Girke T. tgirke/longevityTools. 2019.

4. Hubbell E, Liu W-M, Mei R. Robust estimators for expression analysis. Bioinformatics. 2002;18:1585–92.

5. Ritchie ME, Phipson B, Wu D, Hu Y, Law CW, Shi W, et al. limma powers differential expression analyses for RNA-sequencing and microarray studies. Nucleic Acids Res. 2015;43:e47–e47.

6. Gaulton A, Bellis LJ, Bento AP, Chambers J, Davies M, Hersey A, et al. ChEMBL: a large-scale bioactivity database for drug discovery. Nucleic Acids Res. 2012;40 Database issue:D1100–7.

7. Türei D, Korcsmáros T, Saez-Rodriguez J. OmniPath: guidelines and gateway for literature-curated signaling pathway resources. Nat Methods. 2016;13:966–7.

8. Liu A, Trairatphisan P, Gjerga E, Didangelos A, Barratt J, Saez-Rodriguez J. From expression footprints to causal pathways: contextualizing large signaling networks with CARNIVAL. Npj Syst Biol Appl. 2019;5:1–10.

9. Carlson M. org.Hs.eg.db: Genome wide annotation for Human. 2019.

10. Garcia-Alonso L, Ibrahim MM, Turei D, Saez-Rodriguez J. Benchmark and integration of resources for the estimation of human transcription factor activities. bioRxiv. 2018;:337915.

11. Schubert M, Klinger B, Klünemann M, Sieber A, Uhlitz F, Sauer S, et al. Perturbation-response genes reveal signaling footprints in cancer gene expression. Nat Commun. 2018;9:20.

12. Jaeger S, Min J, Nigsch F, Camargo M, Hutz J, Cornett A, et al. Causal Network Models for Predicting Compound Targets and Driving Pathways in Cancer. J Biomol Screen. 2014;19:791–802.

13. Bradley G, Barrett SJ. CausalR: extracting mechanistic sense from genome scale data. Bioinformatics. 2017;33:3670–2.
